# Beyond ectomycorrhizal bipartite networks: projected networks demonstrate contrasted patterns between early- and late-successional plants in Corsica

**DOI:** 10.3389/fpls.2015.00881

**Published:** 2015-10-20

**Authors:** Adrien Taudiere, François Munoz, Annick Lesne, Anne-Christine Monnet, Jean-Michel Bellanger, Marc-André Selosse, Pierre-Arthur Moreau, Franck Richard

**Affiliations:** ^1^UMR 5175, CEFE – CNRS – Université de Montpellier – Université Paul Valéry Montpellier – EPHE – INSERMMontpellier, France; ^2^UM2, UMR AMAPMontpellier, France; ^3^French Institute of PondicherryPondicherry, India; ^4^CNRS, LPTMC UMR 7600, Université Pierre et Marie Curie-Paris 6, Sorbonne UniversitésParis, France; ^5^CNRS, IGMM UMR 5535, Université de MontpellierMontpellier, France; ^6^CNRS, Muséum National d’Histoire Naturelle, UMR 7205, Origine, Structure et Evolution de la BiodiversitéParis, France; ^7^Département de Botanique, Faculté des Sciences Pharmaceutiques et Biologiques, Université LilleLille, France

**Keywords:** bipartite networks, projected networks, host-specificity, ecological strategies, Mediterranean forests, ectomycorrhiza, ecological mycorrhizal network

## Abstract

The ectomycorrhizal (ECM) symbiosis connects mutualistic plants and fungal species into bipartite networks. While links between one focal ECM plant and its fungal symbionts have been widely documented, systemic views of ECM networks are lacking, in particular, concerning the ability of fungal species to mediate indirect ecological interactions between ECM plant species (projected-ECM networks). We assembled a large dataset of plant–fungi associations at the species level and at the scale of Corsica using molecular data and unambiguously host-assigned records to: (i) examine the correlation between the number of fungal symbionts of a plant species and the average specialization of these fungal species, (ii) explore the structure of the plant–plant projected network and (iii) compare plant association patterns in regard to their position along the ecological succession. Our analysis reveals no trade-off between specialization of plants and specialization of their partners and a saturation of the plant projected network. Moreover, there is a significantly lower-than-expected sharing of partners between early- and late-successional plant species, with fewer fungal partners for early-successional ones and similar average specialization of symbionts of early- and late-successional plants. Our work paves the way for ecological readings of Mediterranean landscapes that include the astonishing diversity of below-ground interactions.

## Introduction

Evaluating the extent and functions of the ecological links that soil biota create among terrestrial plants is a fascinating challenge in ecology. In temperate and boreal forests, ectomycorrhizal (ECM) symbiosis ecologically binds together 3% of terrestrial plant species and more than 6000 filamentous fungal species. Below-ground mycelia directly connect short roots to soil resources, and provide pathways for reciprocal nutrient fluxes and water exchanges among associated individuals (Smith and Read, 2008). ECM symbiosis contributes to indirect interactions among trees through shared fungus partners (Bingham and Simard, 2012), and these interactions facilitate seedling establishment (Richard et al., 2009) and species coexistence (Selosse et al., 2006) in plant communities. ECM symbiosis thereby contributes to plant community dynamics during both primary (Nara, 2006) and secondary ecological succession (Simard et al., 1997; Richard et al., 2009; van der Heijden and Horton, 2009; Bingham and Simard, 2012).

One of the most striking and consistently observed properties of ECM symbioses is the variation over three orders of magnitude of the number of ECM fungal species associating with a plant species, ranging from a few fungal species associating with *Sarcodes sanguinea* and *Neottia nidus-avis* (Kretzer et al., 2000; Selosse et al., 2002) to over 1800 for *Pseudotsuga menziesii* (Molina et al., 1992).

Ectomycorrhizal fungal species also display large variation in the number of associated plant species, e.g.*, Cenococcum geophilum* and *Laccaria amethystina* have been found on most European ECM trees (Horton and Bruns, 2001; Roy et al., 2008) whereas *Alpova alpestris* only associates with one tree species in the genus *Alnus* (Moreau et al., 2011). Phylogenetically constrained interactions have been shown in some specialized lineages, e.g.*, Suillus* sp. associate only with Pinaceae, *Leccinum* with Betulaceae/Salicaceae, and *Alnicola* with *Alnus* (Wu, 2000; den Bakker et al., 2004; Rochet et al., 2011). Hereafter, specialism and generalism are used *sensu* Öpik and Moora (2012), i.e., referring to the ability of organisms to associate with large (interaction generalists; see Glossary in **Box Box 1**) or small (interaction specialists) numbers of partners.

**Box 1. Glossary.**

**FIGURE 1:**
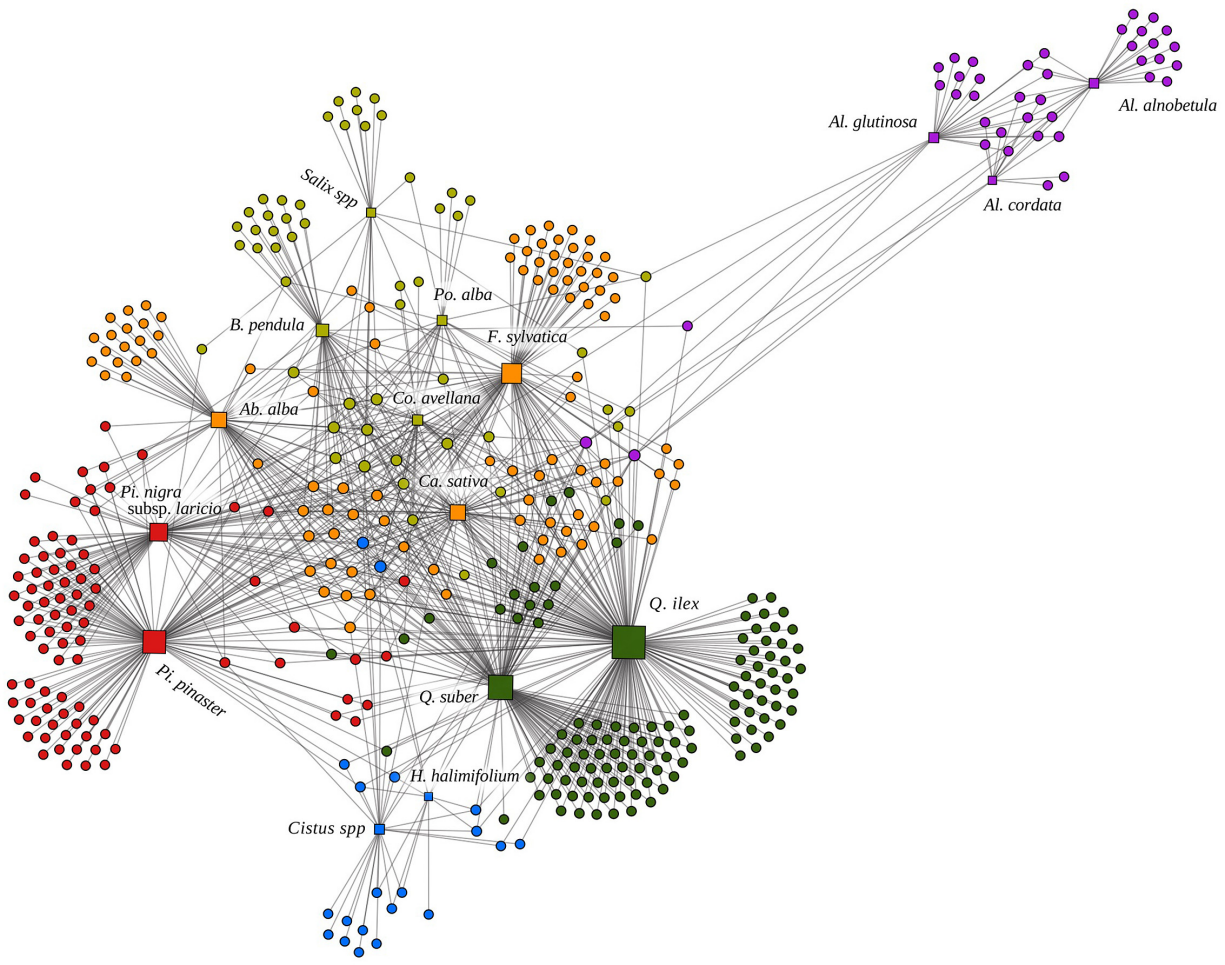
**Representation of the Corsican ectomycorrhizal (ECM) bipartite network based on the algorithm Atlas 2 under the software GEPHI (Bastian et al., 2009).** Squares and circles represent interacting plant and fungal species, respectively. Links indicate plant–fungal interactions. The size of the squares is proportional to the degree of the plant species. Six modules based on Netcarto software (Guimerà and Amaral, 2005) are indicated by different colors. The following abbreviations indicate plant genera: Ab.: *Abies*; Al.: *Alnus*; B.: *Betula*; Ca.: *Castanea*; Co.: *Corylus*; F.: *Fagus*; H.: *Halimium*; Pi.: *Pinus*; Po.: *Populus*; Q.: *Quercus*.

**FIGURE 2:**
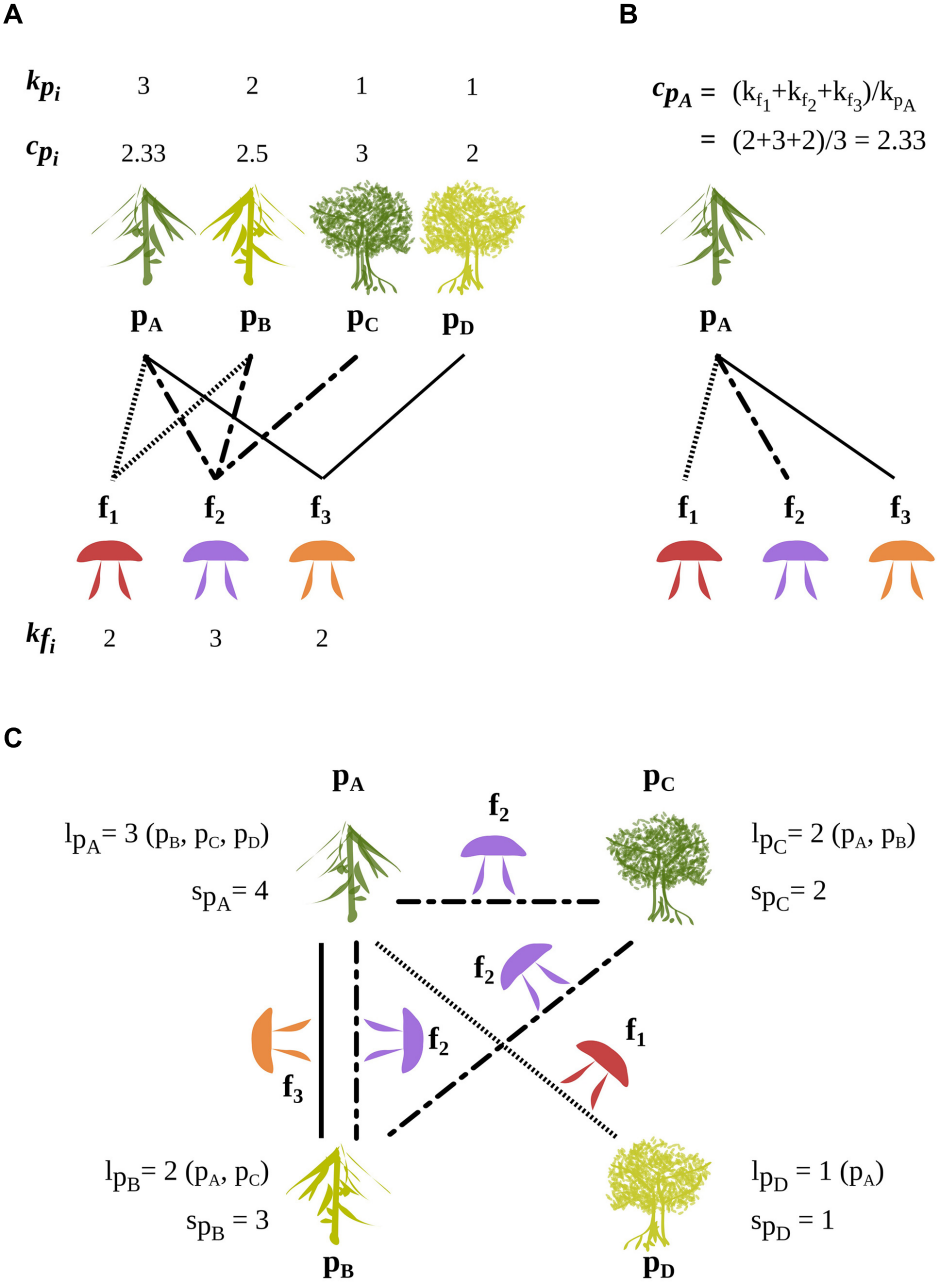
**A schematic bipartite network of four ECM plant species (p_A_, p_B_, p_C_, p_D_) and their three ECM fungal symbionts (f_1_, f_2_, f_3_).** The degree in the bipartite network is *k_p_i__* for a plant species *p_i_* and *k_f_j__* for a fungal species *f_j_*. The average degree of the fungal partners of a given plant species *p_i_* is *c_p_i__*. **(A)** Displays the degrees of plant and fungal species and the average degree of the fungal partners of each plant species. **(B)** Illustrates the detailed calculation of *c_p_A__*. **(C)** Illustrates the plant projected network composed of indirect (two-step) links through fungi, i.e., plant–plant links mediated by shared fungal symbionts. The number of plant species linked to a given plant species through s*_p_*_i_ fungi-mediated links is l*_p_i__*. The numbers of indirect (two-step) links between a given plant species p_i_ and the other plant species is s*_p_*_i_. In all panels, dotted, broken and continuous lines show plant–plant links made by the fungal species f_1_, f_2_, and f_3_, respectively. The roles played by plants and fungi can be inverted to analyze the network from a fungal perspective.

**Bipartite network:** a network linking two distinct sets of nodes (with no links among nodes of the same set). **Figures 1** and **2A** illustrate the ectomycorrhizal (ECM) ecological network linking plant species with fungal species. Bipartite networks are also termed 2-mode networks or bimodal networks.

**Common mycorrhizal network (CMN):** below-ground network where fungal mycelia physically connect roots of different plant individuals.

**Degree (*k*):** in network terminology the degree *k*_n_ of a node *n* is the number of links it has established with other nodes. Here, the degree of a species (either a fungal or a plant species) corresponds to the number of its symbiotic partners. It measures its interaction specialization.

**Ecological mycorrhizal network:** mutualistic interaction network linking together plant and fungal entities (e.g., individuals, populations, species) able to establish a mycorrhizal connection in at least one ecological context and during one ontological stage. The ecological ECM network studied here at the species level (**Figure 1**) is qualitative (binary links) and only informs on the potentiality of two species to interact.

**Interaction specialization:** tendency to interact with few or lot of partners. A species that interacts with many species (high degree *k*) is termed an **interaction generalist** and a species that interacts with few species (low degree *k*) is termed an **interaction specialist**. In the case of the ECM ecological network, fungal interaction specialization is often called host-specificity of the fungal species in mycological literature.

**Modular network:** a modular network is made of subsets of nodes highly connected between them and poorly connected to others. These subsets are called modules or clusters. Modularity in an ecological network may reflect ecological (e.g., spatial or successional position for the plant species) and evolutionary (e.g., coevolution) processes.

**Partner specialization (*c*):** for a focal node *n*, we defined *c_n_* as the mean degree of its partners (its direct neighbors in the bipartite network), where the mean is taken over the set of its partners. Here, the partner specialization of a focal plant species is the mean number of host plant species of its fungal symbionts (**Figure 2A**). The average of *c_n_* over plant species of same bipartite degree recovers the standard bipartite degree correlation (sometimes termed connectivity correlation).

**Projected degree (*l*):** the projected degree *l_n_* of a node *n* of a bipartite network is its degree in the corresponding projected network, that is, the number of nodes of the same set sharing at least one neighbor in the bipartite network (**Figure 2B**). Here, the projected degree of a focal plant species is the number of other plant species sharing fungal partners with it.

**Projected network:** 1-mode network built from a bipartite network, by considering only nodes of one set, and linking two nodes if they share at least one neighbor in the bipartite network (**Figure 2B**); the links of a projected network are also termed indirect links, mediated by nodes of the other set. A bipartite network is associated with two projected networks. Here, the plant projected network links ECM plants through their shared fungal partners (Supplementary Figure S10).

**Projected weight (*s*):** we defined the projected weight *s_n_* of a focal node *n* as the total number of indirect, two-step connections to its neighbors in the projected network in its projected network (**Figure 2B**). Here, the projected weight of a focal plant species is the number of fungal species shared with other plants, where each fungal species is counted as many times as it indirectly links the focal species to another plant species. The projected weight of a focal plant species is in general different from its projected degree *l_n_*, but related to its partner specialization *c_n_* and its bipartite degree *k_n_* according to *c_n_= 1+(s_n_/k_n_).*

Demonstrations of the ecological and evolutionary advantages of specialism vs. generalism in ECM symbiosis are lacking, for several reasons: most ECM fungal lineages are refractive to *in vitro* cultivation, fungal species may appear less host-specific than they really are due to cryptic diversity, and determinants of fungal host-specialization are still controversial (Hawksworth, 2001; Bruns et al., 2002). It is commonly accepted that generalism may enable ECM plants to extend the habitats in which they can establish as seedlings (Bruns et al., 2002; Botnen et al., 2014). Thus, generalist plants may gain access to a greater reservoir of compatible ECM inoculum and may therefore establish more easily than specialist plants. The ability of ECM plants to colonize new areas more rapidly may result from plant ability either to associate with numerous distantly related partners (direct plant generalism), or to associate with a set of fungi that do so (indirect plant generalism through fungal generalism). During the first steps of ecological succession, generalism may primarily drive vegetation dynamics. Early-successional plants first establish in newly available habitats through fungi-mediated facilitation processes (e.g., Nara, 2006), while late-successional tree species colonize (e.g., Selosse et al., 2006; Richard et al., 2009) and outcompete (Bruns et al., 2002) in pioneer vegetation through facultative epiparasitism. At the end of the succession, ECM communities are hyper-diverse assemblages classically associated with long-lived late-successional tree species in old stands (Visser, 1995; Dahlberg et al., 1997; Smith et al., 2002; Richard et al., 2005; Walker et al., 2005). It has been hypothesized that this pattern of fungal diversity enrichment with forest aging may be driven by a late accumulation of host-specific fungi (Last et al., 1983; Smith et al., 2002; Twieg et al., 2007).

Two questions arise. First, is there a trade-off between the number of fungal partners and their specialization at the plant species level? Second, do early- and late-successional plant species differ in their association patterns, with late-successional species accumulating more specialized fungal species than early-successional plants? We here explore these questions by analyzing the correlation between the number of symbionts of a plant species and the average specialization of these symbionts, in regard to the successional status of the plant species in Corsica.

The analysis of bipartite interaction networks has provided important insights into ecological (Bascompte and Jordano, 2007; Chagnon et al., 2014) and evolutionary questions (Ives and Godfray, 2006; Krasnov et al., 2012). Analyses have demonstrated that the topology of the network depends on whether the interactions are mutualistic or antagonistic (Thébault and Fontaine, 2010). To date, most research on bipartite ecological networks has focused on plant-pollinator or plant–herbivore interactions (Thébault and Fontaine, 2010). The structure of local plant–fungi bipartite networks has been investigated at the species level in the context of endomycorrhizal (Chagnon et al., 2012; Montesinos-Navarro et al., 2012; Öpik and Moora, 2012) and orchid mycorrhizal associations (Martos et al., 2012). In the context of the ECM symbiosis, ever-increasing information is available on below-ground associations to inform the links between ECM fungal and plant species. However, studies examining these interactions in a network perspective are lacking (but see Bahram et al., 2014). Moreover, most studies have considered the ECM community as a static biotic component of the plant’s ecological niche.

We here argue that investigating the structure of ECM interspecific networks is a path to explore the ecological role of ECM symbiosis at a systemic level. We have analyzed an ECM ecological bipartite network (see Glossary) constructed from a unique qualitative dataset that exhaustively assembled field and molecular records on ECM plant–fungi associations in the island of Corsica, France. ECM links between plant and fungal species are considered at the scale of the whole island. More specifically, our aim was to understand the potential of ECM symbiosis for creating fungi-mediated ecological interactions (e.g., facilitation or competition) between plant species, and reciprocally between ECM fungal species by means of shared host plants. These indirect interactions among species of the same kind (plants vs. fungi) mediated by species of the other kind constitute *projected networks* (see Glossary; Latapy et al., 2008; Nacher and Akutsu, 2011).

Our study investigated three main questions. First, whether there is a trade-off between the number and the specialization of fungal species associated with a plant species; second, whether plant species associated with fewer fungal species in the bipartite network are linked to fewer plant species in the projected network; and finally, when the ecological strategies of plants are considered, whether early- and late-successional species display different interaction patterns, in either the bipartite network or the projected plant network.

## Materials and Methods

### Study Area

We assembled and analyzed a database on plant species and their associated ECM fungal species all over the Mediterranean island of Corsica (Conservatoire Botanique National de Corse, unpublished data; data are given in Supplementary Table S1, references in Supplementary References S2, detailed description of the methods in Supplementary Methods S3, and the workflow of data filtering in Supplementary Figure S4). The island covers 8681 km^2^ of mountainous territory. It is the sole island in the Mediterranean basin presenting large surfaces of well-preserved native ECM forests (Quézel and Médail, 2003; Richard et al., 2005). The distribution and numbers of plant species and ECM fungal species are exceptionally well-known after over a century of intensive botanical and mycological surveys (Jeanmonod and Gamisans, 2007).

#### Plant Species Included in the Analysis

We constructed a network based on all known ECM plant species present in Corsica (Gamisans, 1991; Jeanmonod and Gamisans, 2007) excluding recently introduced species (*Eucalyptus globulus*, *Larix decidua*, *P. menziesii*, and *Populus nigra*). We also excluded mycoheterotrophic and mixotrophic plants (orchids and Pyroleae; Selosse and Roy, 2009) from the analysis because of the atypical physiology of their relationship with ECM fungi. Eleven additional plant species were excluded from the analysis because of their very low frequency (*Pinus pinea*, *Populus canescens*, *Pinus halepensis*, and *Quercus robur* subsp. *robur*) or because their ECM fungal communities are insufficiently documented (*Arbutus unedo*, *Fumana laevipes*, *Fumana thymifolia*, *Ostrya carpinifolia*, *Populus tremula*, *Quercus humilis* subsp. *Humilis*, and *Quercus petraea* subsp. *petraea*; cf. Supplementary Methods S3).

*Salix* species cover large areas in many locations of the island, where the different species co-occur and their local ECM communities have not been critically analyzed using molecular tools. The same is true for *Cistus* species. Therefore, we conservatively considered each of these genera as a single taxon in the analysis (*Salix* sp. on one hand and *Cistus* sp. one the other hand; cf. Supplementary References S2 and Supplementary Methods S3). The 16 resulting plant taxa (hereafter called plant species) included all the tree species dominating the forest stages of plant ecological successions in Corsica (Gamisans, 1991). They represented all forest ecosystems from sea level (sclerophyllous oak forests) to the upper altitudinal tree limit.

#### Fungal Species Included in the Analysis

For macromycetes, 411 species out of 610 ECM fungal species recorded in Corsica (67%, Supplementary Table S1; Supplementary Methods S3) were included in the network analysis. We retained only taxa for which were available (i) a well-resolved taxonomic treatment, and (ii) reliable data on host association within Corsica based on published records (Supplementary References S2).

#### Validation of the Interactions Included in the Analysis

In total, we defined 993 interactions (**Figure 1**) from 61 informative analyzed datasets extracted from either fruitbody surveys or DNA belowground studies (Supplementary References S2). This two sources of information are two complementary views of plant–fungal ECM links (Horton and Bruns, 2001). Briefly, we compiled a large set of publications (both peer-reviewed and ‘gray literature’), books and records from expert field mycologists. The associations of the 411 selected fungal species with the 16 plant species were compiled as follows. To be validated, each association between plants and fungi was ascertained by molecular studies (Richard et al., 2004; Moreau et al., 2011; Rochet et al., 2011; Roy et al., 2013), or by validated observations within a monospecific forest/chaparral stand (one single ECM host) in Corsica, or by both methods. Molecular data were obtained from mycorrhizal sequencing in published studies. Only mycorrhizal root tips that were (i) taxonomically assigned to a given fungal species (blast) and (ii) related to a given plant host were included in the dataset. Herbarium collections (published or not; most came from the Lille University herbarium LIP, France) associated with field observations were systematically used to validate the taxonomic identity of fungal species.

#### Analysis of Bipartite and Projected Networks

We built a bipartite network including the 16 plant species and 411 ECM fungal species (**Figure 1**). The corresponding 16 × 411 binary matrix of association has value 1 at position (*p*, *f*) if fungal species *f* has been reported to be an ECM symbiont of plant species *p*, and 0 otherwise (Supplementary Table S1). Following network theory terminology, the number of ECM fungal species that are linked with a given plant species *p* is called its bipartite degree *k_p_* (**Figure 2**). The number of plant species that are linked with a given fungal species *f* is called its bipartite degree *k_f_*. Low *k* values characterize interaction-specialist species (*sensu* Öpik and Moora, 2012), while high *k* values characterize interaction-generalist species. Hereafter, plant specialization (generalism vs. specialism) will be used to refer to the realized biotic part of plant niche, and not to any theoretical niche of ECM plants in the Corsican region. The fungal species linked with the plant species *p* may also be linked with other plant species, and the number of plant species that are linked through one or more of these shared fungal partners is called the projected degree of the plant species, *l_p_*. These links constitute the projected network of plant species (or “plant projected network”). We likewise defined the projected degree of a fungal species, *l_f_*, as the number of associated fungal species in the projected network, i.e., linked through plant species to the focal fungal species *f* (**Figure 2**).

A plant species can interact with another plant species *via* a single fungal species or *via* several ones. The number of fungal species linking two plant species is a measure of the strength of their association in the projected network. The total number of indirect (two-steps) links of the plant species *p* to other plant species is called its weight *s_p_* in the plant projected network (links between plant species in **Figure 2C**). Likewise, the total number of plant-mediated links of a fungal species *f* to other fungal species is its weight *s_f_* in the fungal projected network.

While *l_p_* represents the number of plant species to which *p* is linked in the plant projected network, *s_p_* represents the total number of links established *via* fungal species with these plant species. The contribution to *s_p_* of a plant species is simply the number of ECM fungal species that it shares with *p.* Therefore, the ratio of *s_p_* to *l_p_* measures in an integrated way the redundancy of fungal species in establishing links between *p* and other plant species.

If the number of links of a given plant species to other plant species is selected for, then we may expect a trade-off for the plant between its specialization (value of *k_p_*) and the specialization of the fungi (value of *k_f_*) with which it associates. Indeed, a plant species associated with many specialized fungal species (high *k_p_* and partners with low *k_f_*) may be as well-linked (same *s_p_*) with as many other plant species (same *l_p_*) as a plant species associated with few generalist fungal species (low *k_p_* and partners with high *k_f_*). To explore this question, we introduced an additional quantity, *c_p_*, measuring the average specialization of the fungal partners of a given plant species *p.* It is defined as the mean degree (in the bipartite network) of the fungal partners of the plant species, *p*, where the average runs over the set of these fungal partners. We then devised two null models to analyze the relationships between *c_p_* and *k_p_* (see below). A summary of the different network-related notions and their notation is given in the Glossary (**Box Box 1**).

#### Null Models

Null models were used to simulate random situations sharing some minimal constraints with the actual network by randomizing links among plant and fungi. Deviations of observed network statistics from the statistics measured in these random situations inform on whether species are more generalist or specialist than expected by chance, or whether they are associated with partners that are themselves more generalist or specialist than expected by chance, given the specified constraints.

First we devised a simple random model (null model 1) considering independently each pair of plant and fungal species and drawing a link with a probability equal to the density of links (actual number of links divided by the maximal number of links given the number of plant and fungal species) observed in the real network. As a consequence, the mean values of plant and fungal bipartite degrees are the same in this null model 1 and the real network.

**FIGURE 3:**
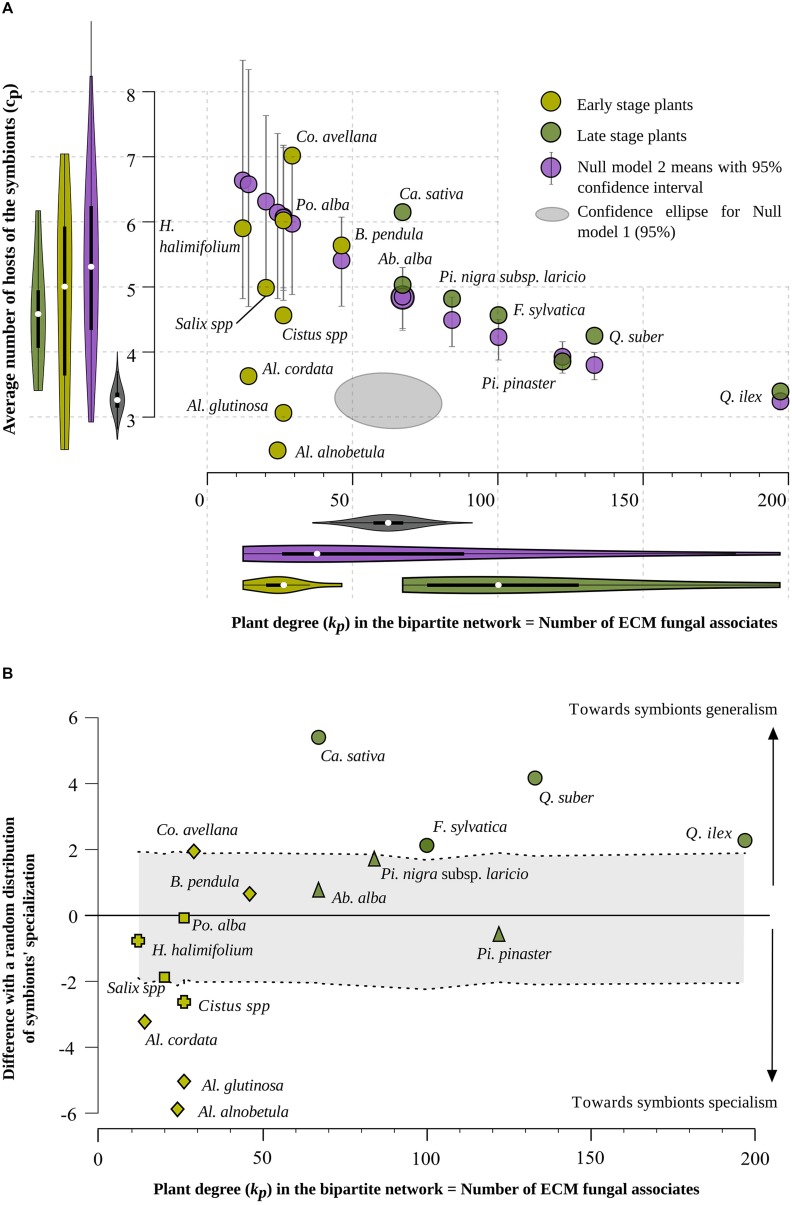
**(A)** Distribution of 16 ECM plant species according to their degree in the bipartite network, *k_p_*, i.e., the number of their fungal partner species (X axis), and the average number of host species (i.e., average specialization) of their symbionts, *c_p_* (Y axis). Light green and dark green dots correspond to early-stage and late-stage plant species, respectively. Gray ellipses display the 95% confidence intervals for null model 1. The null model 1 consists of a simple random model considering independently each pair of plant and fungal species and drawing a link with a probability equal to the density of links (actual number of links divided by the maximal number of links given the number of plant and fungal species) observed in the real network. Purple dots show positions of plant species in the null model 2. The null model 2 is based on a random distribution of the links in the bipartite network, at fixed bipartite degrees *k_p_* or *k_f_* for all partners. Bars indicate 95% confidence intervals. **(B)** Distribution of 16 ECM plant species according to their degree in the bipartite network, *k_p_*, i.e., the number of their symbiont species (X axis) and the standardized deviation [standard effect size (SES)] of the average number of host species of their symbionts, *c_p_*, with respect to its value in null model 2. A total number of 999 randomized networks were simulated to establish the null situation with which the actual *c_p_* values were compared. For plant species located between the two dotted curves, the observed situation does not differ from a random distribution of their links, whereas a plant species located above the upper dotted line shows an excess of generalist species among its associated ECM fungal species, and a plant species situated below the lower dotted line shows a significant excess of specialist symbionts. The following abbreviations and signs are used to indicate plant genera and families: Ab.: *Abies*; Al.: *Alnus*; B.: *Betula*; Ca.: *Castanea*; Co.: *Corylus*; F.: *Fagus*; H.: *Halimium*; Pi.: *Pinus*; Po.: *Populus*; Q.: *Quercus*; circle: Fagaceae; triangle: Pinaceae; square: Salicaceae; cross: Cistaceae; diamond: Betulaceae.

The distributions and relationships between *k_p_* and *c_p_* values obtained with this null model were compared with the distribution and relationships obtained in another null model, in which the bipartite degree of each plant or fungal species is exactly the same as in the real network, but the links between species were randomized (null model 2). Both null models were generated using the *permat* functions within the R package Vegan with (model 2; method *quasiswap* of the function *permatswap*; Patefield, 1981) or without (model 1; function *permatfull*) preserving column and row sums (Oksanen et al., 2015). Using these two null models, it is possible to identify the correlations between *k_p_* and *c_p_* values which are simply due to the degree distributions in the dataset, i.e., to the distribution of specialism vs. generalism. Comparison of the data with null model 1 allowed detecting whether species bipartite degrees were distributed randomly within the network (Gotelli and Entsminger, 2003). We present our results on partner specialization as a scatter plot, keeping track of the plant species identity and the variance of their partner specialization (**Figure 3A**). Finally, observed values of *c_p_* and *k_p_* were compared with their statistics obtained in the null model 2 in order to detect whether the actual network differs from a random network in which levels of species specialization (values of *k_p_* and *k_f_*) were identical to those observed in the real data.

Given that the association matrix is highly asymmetric (16 plants ^∗^ 411 fungi), our analysis focused on a plant perspective as more information (more potentially associated partners) is available for each plant, allowing a more powerful statistical analysis. The fungal-perspective analysis is presented in Supplementary Figure S5.

#### ECM Association Patterns and Ecological Strategy of Plant Hosts

Early- stage vs. late-stage status of each plant species was determined according to reference work on their ecology in Corsica (Jeanmonod and Gamisans, 2007; Rameau et al., 2008). Accordingly, all species belonging to the Salicaceae, Betulaceae, and Cistaceae were classified as early-stage plants, while all species belonging to the Pinaceae and Fagaceae were classified as late-stage plants. We compared the degrees of plant species in the bipartite network (*k_p_*), their weights in the projected network (*s_p_*) and average numbers of hosts of their fungal symbiotic species (*c_p_*) in the sets of early-stage and late-stage plant species, using Mann–Whitney non-parametric tests. We measured modularity using the simulated annealing algorithm implemented in Netcarto (Guimerà and Amaral, 2005). This software also simulates random graphs with the same degree distribution as the original network to test for modularity significance (Guimerà et al., 2004).

## Results

### Relationships between the Number of ECM Partners and their Specialization

Among the 16 plant species, association patterns varied from species-poor (low *k_p_* values) fungal communities associated with Betulaceae (*Alnus, Betula, Corylus*, whose number of associated ECM fungal species ranged from 14 to 46) and Cistaceae (*Cistus* and *Halimium* with *k_p_* = 26 and *k_p_* = 12, respectively) to rich ECM fungal assemblages (high *k_p_* values) associated with *Quercus suber* and *Quercus ilex* evergreen oaks (respectively 133 and 197 ECM linked fungal species; **Figure 3A**). There was no significant correlation between the bipartite degree k_p_ of a plant species and the interaction specialism c_p_ (average number of host species) of its associated fungal species (Spearman non-parametric rank correlation test, *p* = 0.71; **Figure 3A** green points).

The variances of the plant (*k_p_*) and fungal (*k_f_*) bipartite degrees were much higher in the real network than in the simple random model (null model 1; **Figure 3A** and Supplementary Figure S5 for *k_f_*). We then constrained the null model so that the degree of each plant (*k_p_*) and fungal species (*k_p_*) is the same as in the real network (null model 2, 999 permutations). The simulation revealed a strong negative correlation between plant degree (*k_p_*) and mean interaction specialism of fungal partners (*c_p_*) (**Figure 3A** purple points). Hence, *k_p_* and *k_f_* values impose a constraint on the structure of the network which *a priori* results in a strong negative correlation between number of associated fungal species and degree of specialization of these fungal species (**Figure 3A** purple points). In the real network, the absence of correlation between the bipartite degree *k_p_* of a plant species and the average number of hosts *c_p_* of its associated fungal species is unexpected given the distribution of *k_p_* and *k_f_* values.

Comparing the real network and the null model with constrained *k_p_* and *k_f_* values (null model 2) shows that the value of *c_p_* deviates from the null model for more than half of the species (**Figure 3B**). Four plant species (*Cistus* sp.*, Alnus glutinosa, Alnus alnobetula* subsp. *suaveolens*, and *Alnus cordata*) host significantly more specialist fungal species than would be expected by chance (given the distributions of degrees; *p*-values < 0.025 using the null model 2 for these species) while the four Fagaceae species (*Q. ilex*, *Q. suber, Fagus sylvatica*, and *Castanea sativa*) and *Corylus avellana* host significantly more generalist fungal species than expected by chance (**Figure 3B**; *p*-values < 0.025 using the null model 2 for these species).

### Projected Networks of ECM Plants

Ten plant species are linked to all other plant species (*l_p_* = 15; **Table 1**). Indeed, the number of plant species to which a plant species is linked in the projected network varies from 11 (*A. cordata* and *A. alnobetula*) to all other 15 species (*A. glutinosa* and all species belonging to Cistaceae, Pinaceae, or Fagaceae; **Table 1**). Behind the tendency of all plant species to saturate their projected network, there is wide variation in the number of ECM-mediated links (two-step links) that a given plant species makes with all other plant species (*s_p_* ranging from 36 ECM-mediated links for *A. alnobetula* to 474 links for *Q. ilex*; Supplementary Figure S6). For instance, 12 ECM partners link *Halimium halimifolium* with all plant species (altogether mediating 59 two-step links), so that the neighborhood of *Halimium halimifolium* in the projected network includes all the plant species of the present survey. In contrast, *Betula pendula* symbiotic links do not saturate the plant–plant projected network even though its 46 associated ECM fungal species establish 214 fungi-mediated links.

**Table 1:** Network properties of the 16 ECM plant taxa.

	*H. halimifolium*	*Al. cordata*	*Salix* sp.	*Al. Alnobetula*	*Cistus* sp.	*Al. glutinosa*	*Po. alba*	*Co. avellana*	*B. pendula*	*Ca. sativa*	*Ab. alba*	*Pi. nigra* subsp. *laricio*	*F. sylvatica*	*Pi. pinaster*	*Q. suber*	*Q. ilex*
k_p_	12	14	20	24	26	26	26	29	46	67	67	84	100	122	133	197
c_p_	5.92	3.64	5	2.5	4.58	3.08	6.04	7.03	5.65	6.16	5.04	4.83	4.58	3.87	4.26	3.41
l_p_	15	11	13	11	15	15	13	13	13	15	15	15	15	15	15	15
s_p_	59	37	80	36	93	54	131	175	214	346	271	322	358	350	434	474
n_hs_	1	2	8	13	7	8	18	3	14	1	18	2	29	27	1	39

*k_*p*_: degree in the bipartite network, i.e., the number of their fungal partner species; *c_*p*_*: average number of host species of their symbionts; *l_*p*_*: degree of plant species in their projected network; *s_*p*_*: total number of links of a plant species within its projected network; *n_*hs*_*: number of hyper-specialists, i.e., strictly associated fungal taxa. See Glossary for more complete definitions of network metrics*.

*The following abbreviations were used to indicate plant genera: Ab.: *Abies*; Al.: *Alnus*; B.: *Betula*; Ca.: *Castanea*; Co.: *Corylus*; F.: *Fagus*; H.: *Halimium*; Pi.: *Pinus*; Po.: *Populus*; Q.: *Quercus**.

Endemic alders, *A. cordata* and *A. alnobetula*, with *l_p_* = 11 plant partners through *s_p_* = 37 and 36 fungi-mediated links respectively, interact with slightly less plant species than the wide-ranging *A. glutinosa* (*l_p_* = 15 through *s_p_* = 54 links; **Table 1**). In our analysis, these two endemic alders establish no ECM fungi-mediated links with *Betula*, *Corylus*, *Populus*, or *Salix*.

There is wide variation in the number of ECM-mediated links (two-step links) that a given plant species makes with all other plant species (*s_p_* values, taken as the weight of the plant species in the projected network), ranging from 36 ECM-mediated links for *A. alnobetula* to 474 links for *Q. ilex* (Supplementary Figure S6).

The redundancy *l_p_*/*s_p_* (ratio of the projected degree *l_p_* to the number of comprised links *s_p_*) ranges from 11/36 = 0.306 for *A. alnobetula*, to 15/474 = 0.032 for *Q. ilex* (**Table 1**). These results indicate a lower redundancy of the ECM-mediated links of *A. alnobetula* to other plant species as compared to *Q. ilex*.

### ECM Associations along Ecological Succession

**FIGURE 4:**
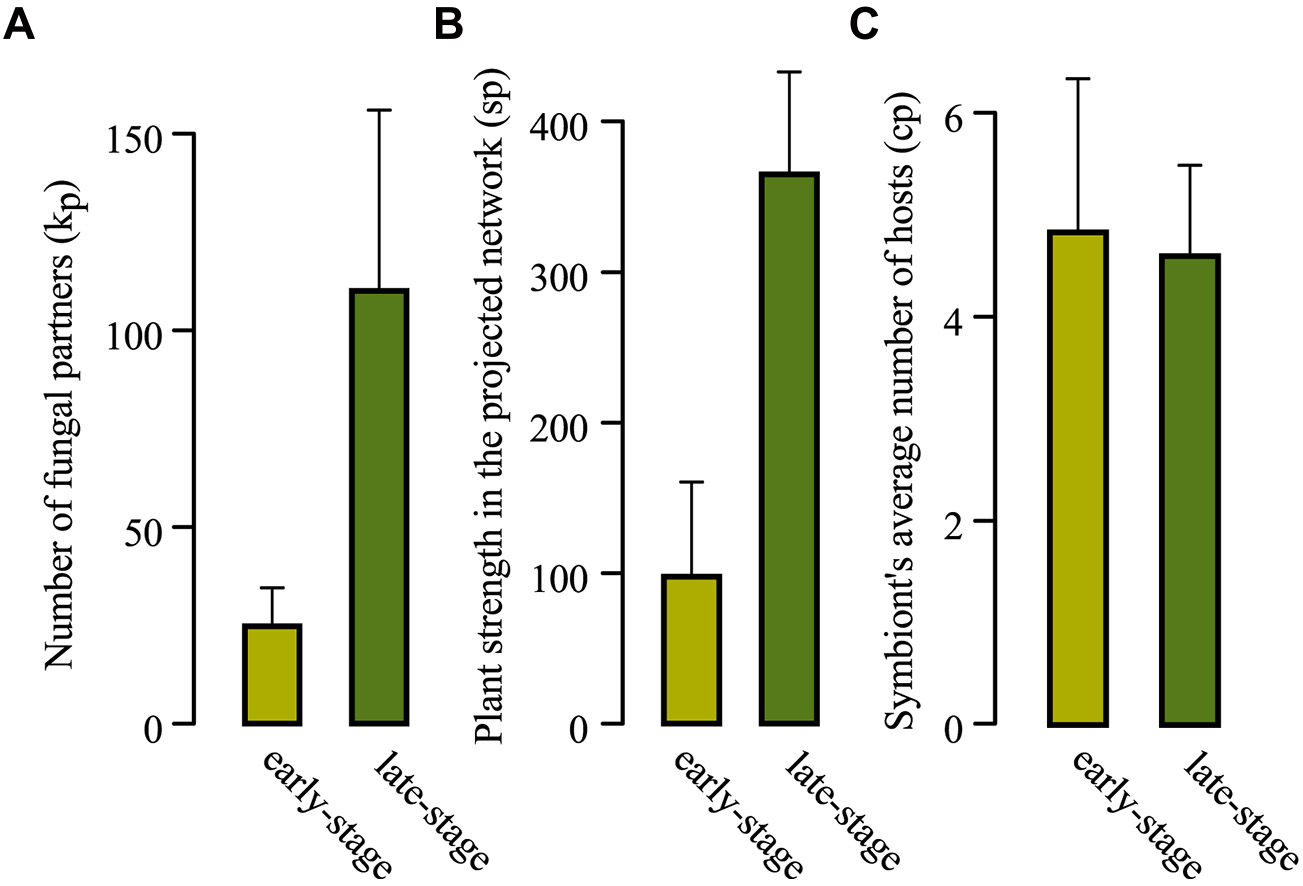
**(A)** Comparison of the average plant bipartite degree (average of *k_p_*), **(B)** average number of fungi-mediated links (average of *s_p_*), and **(C)** average mean number of host species of fungal symbionts (average of *c_p_*) over the set of early-stage plant species (light green) and the set of late-stage (dark green) plant species. Bars indicate standard errors.

Early-stage vs. late-stage plant species displayed contrasted distributions of their numbers of associated fungal species (**Figure 3** and **Table 1**, with a maximal degree *k_p_* = 46 for the set of early-stage species and a minimal degree *k_p_* = 67 for the set of late-stage species). On average, early-stage plant species associated with five times fewer fungal species (mean *k_p_* = 24.78 for early-stage vs. 110 for late stage plant species; *p* < 10^-4^, Mann–Whitney non-parametric test; **Figure 4A**). They also present five times fewer fungi-mediated links to other plant species in the projected network (mean *s_p_* = 97.67 vs. 365; Mann–Whitney *p* < 10^-4^; **Table 1** and **Figure 4B**) and five times higher *l_p_*/*s_p_* ratios (mean ratio = 0.38 vs. 0.083; Mann–Whitney *p* < 10^-3^). Nevertheless, their associated fungal species did not differ in their partner specialization *c_p_* (average number of host species of its symbionts; mean *c_p_* = 4.83 vs. 4.59; Mann–Whitney *p* = 0.84) from those associated with late-stage plant species (**Figures 3A** and **4C**).

We compared the sharing of ECM symbionts observed in the real system (i) between early- and late-stage plant species, (ii) among early-stage plant species, and (iii) among late-stage plant species, against the sharing calculated in the null model with constrained *k_p_* and *k_f_* values (null model 2). Fewer ECM fungal species (62) than expected by chance (108.52 ± 4.95 under null model 2) interact with both early- and late-stage plant species (*p* < 10^-3^). More ECM fungal species (37) are exclusively associated with early-stage plant species than expected by chance (25.54 ± 4.21 under null model 2; *p* < 10^-3^) given the number of partner species. On the contrary, the number of ECM fungal species (272) exclusively associated with late-stage plant species is close to the value expected by chance (277.21 ± 4.33 using null model 2; *p* = 0.09). These results show that during ecological successions, late-successional vegetation stages accumulate rich ECM communities comprised of specialist fungi that differ in composition compared to the communities associated with early-successional stages (Supplementary Figures S7–S9).

We further used the modularity of the whole network to characterize the sharing of ECM symbionts among early- and late-stage plant species. We detected six modules in a significantly modular system (*M* = 0.458, null model = 0.362 ± 0.002). Three modules included only early-stage plant species (represented in light green, blue and purple in **Figure 1**) and the three others included the late-stage species (represented in orange, red and dark green in **Figure 1**).

**FIGURE 5:**
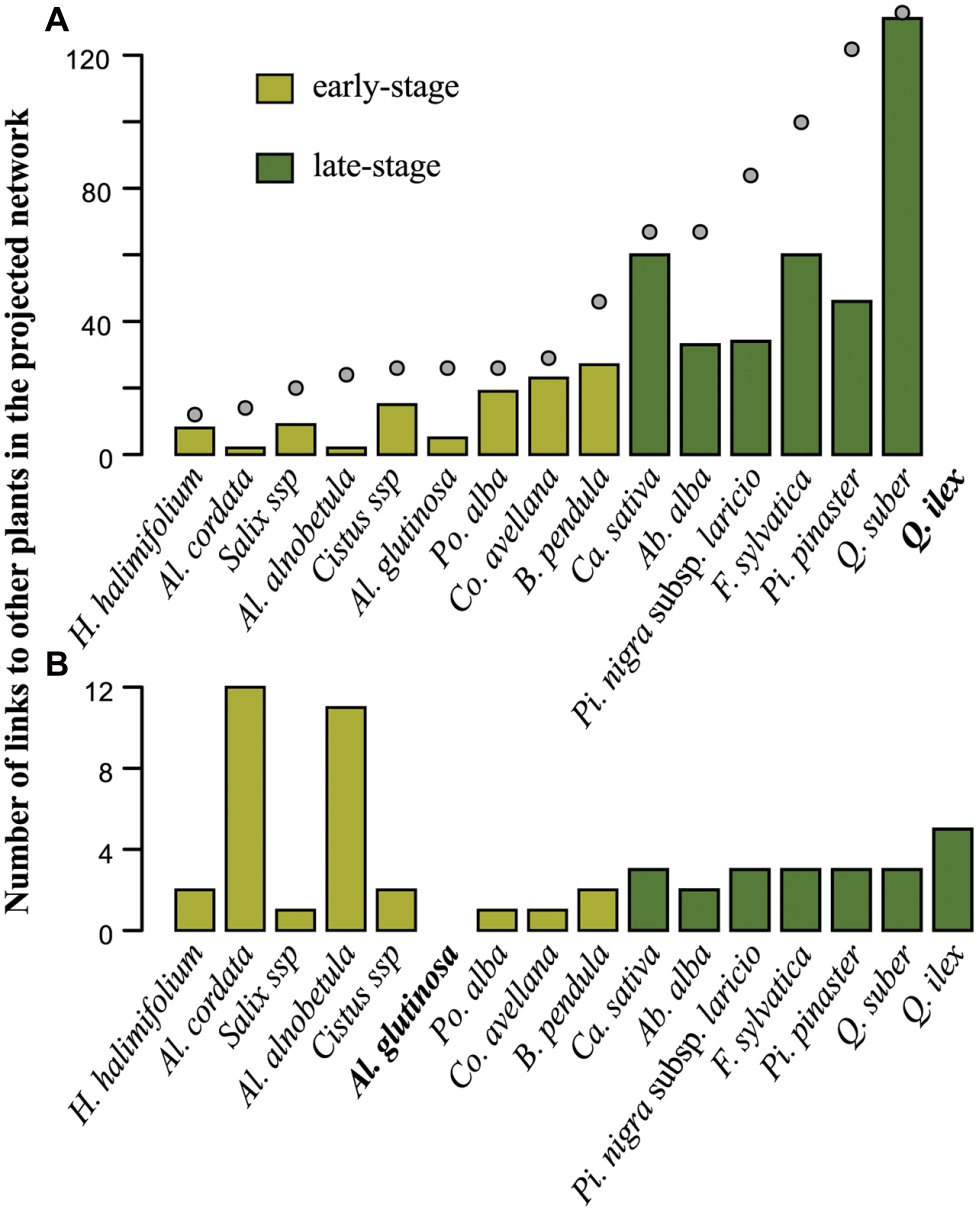
**Number of shared fungal species (in network terminology, number of fungi-mediated links) between **(A)***Quercus ilex* or **(B)***Alnus glutinosa* and other plant species (ordered at increasing number of fungal partners (*k_p_*)).** The color of the bars indicates early-stage (light green) and late-stage (dark green) plants species. **(A)** Gray dots indicate plant degrees (*k_p_*) in the bipartite network (same scale on the vertical axis). The following abbreviations are used to indicate plant genera: Ab.: *Abies*; Al.: *Alnus*; B.: *Betula*; Ca.: *Castanea*; Co.: *Corylus*; F.: *Fagus*; H.: *Halimium*; Pi.: *Pinus*; Po.: *Populus*; Q.: *Quercus*.

Comparing *A. glutinosa* (early-stage) and *Q. ilex* (late-stage) illustrates the contrast between early- and late-stage plant species association patterns (**Figure 5**). Fungi-mediated interactions of *Q. ilex* are (i) quantitatively highly variable (from two links toward *A. cordata and A. alnobetula* to 131 links toward *Q. suber*) and (ii) qualitatively more numerous toward Fagaceae (*Q. suber*, *C. sativa*, and *F. sylvatica* accounted for 52.95% of the 474 indirect links of *Q. ilex*). All but two fungal species associating with *Q. suber* also associate with *Q. ilex* in Corsica (*Inocybe fibrosoides* is strictly associated with *Q. suber* and *Boletus pulverulentus* is strictly associated with *Q. suber* and *C. avellana*). Similarly, *A. glutinosa* presents a high variation in the number of fungi-mediated links toward other plant species, ranging from one (*C. avellana*, *P. alba*, and *Salix* sp.) to 12 (*A. cordata*). Both *A. glutinosa* and *Q. ilex* show higher numbers of fungi-mediated links toward plant species belonging to their own genus (*A. alnobetula* and *A. cordata* for *A. glutinosa*, and *Q. suber* for *Q. ilex*) and, for *Q. ilex*, to its family (*C. sativa* and *F. sylvatica* for *Q. ilex*; **Figures 1** and **5**).

## Discussion

In this paper we analyzed the main properties of the ECM bipartite network linking host plants and fungal species in Corsica and deciphered ECM network structure in an ecological perspective. We did not detect significant correlation between the specialization of plant species and the specialization of their symbionts across the whole plant dataset, but rather observed decoupled patterns of symbiont diversity (number of fungal partners associated with a given plant species) and the specialization of these symbionts (average number of plant partners of these symbionts). Our results show that early- and late-successional plant species share fewer fungal symbionts than expected by chance, with early-successional species presenting fewer links caused by fewer fungal partners than late-successional plant species, and no difference in average specialization of fungal symbionts of early- and late-successional plant species.

### Relationship between the Number and the Specialization of ECM Symbionts

In our analysis, plant species hosting the richest ECM fungal assemblages associate with fungi whose host ranges are similar to those of plants with few fungal partners (**Figure 3A**). In other words, the analysis of the whole dataset shows no trade-off between plant specialization and the specialization of its symbionts. As a consequence, the number of fungal partners of a plant species (*k_p_*) poorly reflects the number of plant species with which it has fungi-mediated indirect interactions (*l_p_*).

### Insights into the Hyper-diversity of Oak Symbionts

The positions of *Quercus* species in both **Figures 3A,B** confirm the well-known hyper-diversity of ECM communities of evergreen oaks (Richard et al., 2004; Morris et al., 2008). Here, we show that the high diversity of fungal symbionts dissimulates contrasted specialization degrees, with few hyper-generalists occurring among a disproportionately high number of interaction-specialist fungal species. Both oak species deviate from the null model by associating with fungi that are less specialized on average than expected by chance (**Figure 3B**). Interestingly, all but two fungal symbionts of *Q. suber* are also associates of *Q. ilex*. Only two of the 133 fungal symbionts of *Q. suber* are host-specific, while 39 species out of the 197 fungal associates of *Q. ilex* are host-specific (**Table 1**). This pattern may result from a mix of phylogenetic and ecological proximity. Indeed, the distribution and the ecological range (including soil chemical conditions and climatic requirements) of *Q. suber* in Corsica tend to be included within those of *Q. ilex* (Gamisans, 1991). This extensive sharing of fungal symbionts between the two oak species results in a low specialization of oak symbionts (low number of single-host fungal species). However, in these hyper-diverse communities, the symbionts are mostly oak specialists that establish few links to tree species belonging to other genera: 57 of the 131 fungal species associated with both oak species are only associated with oaks (Supplementary Figure S10).

### Contrasted Structures of Bipartite and Plant Projected Networks

Our ECM network presents a modular structure (**Figure 1**) due to abundant plant–plant connections at genus (e.g., within *Alnus*, **Figure 5B**), family (e.g., within Fagaceae, **Figure 5A**; see also Supplementary Figure S7) and ecological (early- vs. late-stage, **Figure 4** and Supplementary Figure S8) levels. Our results complement those of previous studies that suggested modularity of below-ground mycorrhizal networks (Chagnon et al., 2012; Martos et al., 2012; Montesinos-Navarro et al., 2012).

The analysis of the structure of plant–plant interaction patterns (Supplementary Figure S10) through compatible fungal species reveals that generalism prevails in the plant projected network. Among the 16 species, 10 of them interact with all the other plant species and only two interact with fewer than 13 plant species (**Figure 3A**; **Table 1**). This tendency to saturate the projected network (Supplementary Figure S10) contrasts with the high heterogeneity in plant degree found in the bipartite network (**Figure 3A**). As a consequence, similar numbers of neighbors in the plant projected network hide highly variable numbers of symbionts (**Table 1**; **Figure 5**). The contrasted patterns observed for *A. glutinosa* and for *Q. ilex* illustrate this point. In our analysis, *A. glutinosa* saturates the plant–plant network based on only 54 fungal-mediated links whereas *Q. ilex* use 474 such links to do so (**Figure 3A**). This result points out the composite nature of *Alnus*-associated communities, which are comprised of (i) fungal symbionts belonging to *Alnus*-associated fungal lineages (e.g., *Alpova*, *Alnicola*), (ii) interaction specialists scattered across distant fungal lineages and (iii) a few generalist fungal species (Rochet et al., 2011; Roy et al., 2013).

Four species, including the three alder species and the *Cistus* sp. group, host more specialized symbionts on average in their projected network than expected by chance. Two of them, the endemic *A. alnobetula* subsp. *suaveolens* and *A. cordata*, displaying the lowest numbers of fungi-mediated links with other plant species in the whole dataset, share no fungal species with four other genera, *Betula*, *Corylus*, *Popula*, and *Salix*. This result may be surprising when we consider the convergent affinity of alders and the four latter genera for hygrophylic habitats in Corsica. These patterns may be partially explained by ecological requirements of *A. alnobetula* subsp. *suaveolens* and *A. cordata*. The first species highly dominates shrubby vegetation above the current altitudinal limit of forests, at high elevation (Jeanmonod and Gamisans, 2007). In these ecosystems, only endomycorrhizal trees (*Acer*, *Sorbus*) and scattered montane ECM species (*Pinus*, *Abies*, and *Fagus*), but no *Betula*, *Corylus*, *Populus* and *Salix*, remain in the landscape (Jeanmonod and Gamisans, 2007). The second *Alnus* species, *A. cordata*, displays (i) a low dependence on water compared to other alders, *Salix* and *Populus*, and (ii) an ability to establish under *Pinus*, *Abies*, *Fagus*, and *Quercus* during secondary successions (Gamisans, 1991; Jeanmonod and Gamisans, 2007). For these two endemic alders, their positions in the projected network indicate an unusual below-groundecology.

### Below-ground Ecological Strategies of ECM Plants

In previously published literature, both the number of associated fungal species and the number of shared fungal species between plant species have been used for evaluating the potential of plants to interact with other plants through ECM fungal networks (Richard et al., 2005; Nara, 2006; Bingham and Simard, 2012). In our study, we did not hypothesize that the existence of a link between two plant species in the projected network induces any effect of one plant species on the local establishment of another. In this regard, our approach strongly differs from individual plant-centered studies investigating physical networks (Common Mycorrhizal Networks; e.g., Nara, 2006). The links we study here encompass a wide spectrum of plant–plant interactions, from the simple ability for a given plant to provide suitable habitats for other plant species through dispersed fungal propagules, to the possibility that roots of co-occurring species are inter-connected through shared mycelia.

A high plant degree (high number of fungal partners) has been hypothesized to increase physical networking and facilitate seedling establishment under pre-established trees in either conspecific (Dickie et al., 2005; Walker et al., 2005; Bingham and Simard, 2012) or mixed species populations (Amaranthus and Perry, 1989; Horton et al., 1999; Selosse et al., 2006) during secondary successions. The number of shared symbionts has been widely used as a proxy of the strength of the fungi-mediated association between pairs of host plants. Based on these ECM community overlaps, previous studies have suggested for instance facilitation of the establishment of oak forests in a Corsican succession process (*Quercus* – *Arbutus*; Richard et al., 2009), facilitation of coexistence of *Quercus* species (Walker et al., 2005), facilitation of presence of a species of *Helianthemum* at the edge of *Quercus* forests (Dickie et al., 2004) or the capacity of *Betula papyrifera* to constitute a nurse plant for *Pseudotsuga menziesii* (Simard et al., 1997; Simard and Durall, 2004; Bingham and Simard, 2012).

Beyond such fungi-mediated interactions between plant species, the ecological and evolutionary implications of the ability of an ECM plant species to interact with a high number of plant species remain undocumented. A saturated ECM projected network may increase fungal inoculum availability by maintaining compatible reservoirs on alternative hosts. This tendency to share symbionts with many other plants may enlarge the biotic component of the plant niche. Specifically, a high degree in the projected network may facilitate seedling establishment in vegetation of various stages and composition. Such plant-mediated facilitation mechanisms may have been particularly favored in Mediterranean ecosystems where summer drought drastically impacts tree recruitment (Bruno et al., 2003; Gómez-Aparicio et al., 2004). Further studies are required to ascertain the place of ECM inoculum-driven processes in facilitation mechanisms (Richard et al., 2009), which may potentially counterbalance local accumulation of pathogens on plant species (the so-called Janzen–Connell effect, already reported for Mediterranean tree species; Steinitz et al., 2011).

For fungal species, the ECM projected network provides a view of the pool of potentially interacting fungal species. As in plant-centered approaches, calculating the relative overlap in host plants between pairs of fungal species (for instance, using Jaccard distances) would provide fungal species attributes. Mediterranean forests are generally dominated by locally monospecific tree stands (Quézel and Médail, 2003). In this context, a high fungal degree (high number of hosts) may be important for broadening the range of forest types where a fungus can establish. Alternatively, constraining environmental conditions in Mediterranean ecosystems may act as primary abiotic filters selecting for both plant and fungal specialists. We note here that *Salix* and *Alnus* species are adapted to hydromorphic soils, where their host-specific fungal partners may exhibit adaptations to this specific abiotic environment.

In our study, ECM association patterns significantly differed depending on the ecological strategy of their plant host. On average, early-stage plants had five times fewer fungal associates than late-stage ones (**Figure 4**). Our data do not support the hypothesis that early-stage plants differ in the specialization of their symbionts from late successional species, but suggest that early-stage plants associate with significantly fewer ECM fungal species than late-stage plants. Additionally, long-lived late-successional plant species that dominate forest ecosystems for centuries allow a lasting fungal recruitment, which may entail the accumulation of pioneer ECM species. Interestingly, our dataset encompasses mostly species belonging to late-successional fungal genera (*Russula*, *Boletus*, *Amanita*, various “Aphyllophorales” including Bankeraceae and Hydnaceae; Last et al., 1983) that increase in relative abundance with tree aging (Horton and Bruns, 2001). Our results thus suggest that the cumulated effect of late-successional host-plant establishment (above-ground dynamics driven by plant ecological strategies) and host aging-related accumulation of late-successional stage fungal species (below-ground dynamics driven by fungal ecological strategies) lead to the hyper-diversity of ECM communities in mature forests, with no effect on the average specialization of the below-ground ECM diversity (Twieg et al., 2007).

## Conclusion

To the best of our knowledge, our work documents for the first time the below-ground fungal counterpart of the ecological strategies of ECM host plants. Despite the unprecedented rhythm of research describing ECM communities worldwide, their role in plant community dynamics in general, and in host coexistence processes in particular, remains largely unexplored, mainly because most ECM fungi are not cultivable. We assembled a large qualitative dataset to propose a systemic view of the interactions established by the tremendous ECM fungal diversity at the scale of a large Mediterranean island. The ECM symbiosis shapes ecological and evolutionary-based interaction modules, and can help us understand the below-ground niche differences between early- and late-stage plants. The huge diversity of symbionts contributes to saturate plant–plant networks through highly variable numbers of fungi-mediated interactions between plants. Assembling quantitative data of below-ground plant–fungi interaction is crucial to grasp the ecological dimension of these contrasted patterns.

## Author Contributions

AT, FM, AL, FR originally formulated the idea, developed the methodology and performed statistical analyses. A-CM, J-MB, P-AM, FR generated data. AT, FM, P-AM, AL, FR wrote the initial manuscript. AT, FM, AL, A-CM, J-MB, M-AS, P-AM, and FR contributed to the final manuscript.

## Conflict of Interest Statement

The authors declare that the research was conducted in the absence of any commercial or financial relationships that could be construed as a potential conflict of interest.
