# Supplementary material for: Beyond ectomycorrhizal bipartite networks: projected networks demonstrate contrasted patterns between early- and late-successional plants in Corsica
Source: Front Plant Sci. 2015 Oct 20;6:881. doi: 10.3389/fpls.2015.00881 (PMC4612159; doi:10.3389/fpls.2015.00881)
Supplement: Supplementary file 1 [file Data_Sheet_1.DOCX]

***Supplementary Material***

**Beyond ectomycorrhizal bipartite networks: projected networks demonstrate contrasted patterns between early- and late-successional plants in Corsica.**

**A. Taudiere ^1*^, F. Munoz ^2^, A. Lesne ^3^, A.-C. Monnet ^1^, J.-M. Bellanger ^1^, M.-A. Selosse ^4^, P.-A. Moreau ^5^, F. Richard ^1^**

**Affiliation and Address:**

^1^ UMR 5175 CEFE – CNRS - Université de Montpellier - Université Paul Valéry Montpellier - EPHE - INSERM, Campus CNRS, 1919 Route de Mende, F-34293 Montpellier, France

^2^ UM2, UMR AMAP, Boulevard de la Lironde, 34398 Montpellier Cedex, France & French Institute of Pondicherry, 11 Saint Louis Street, Pondicherry 605001, India

^3^ CNRS, LPTMC UMR 7600, Université Pierre et Marie Curie-Paris 6, Sorbonne Universités, 4 Place Jussieu, F-75252 Paris Cedex 05, Paris, France & IGMM UMR 5535, CNRS Université de Montpellier, 1919 Route de Mende, F-34293 Montpellier, France

^4^ Muséum National d’Histoire Naturelle, UMR CNRS 7205, Origine, Structure et Evolution de la Biodiversité, 16 rue Buffon, CP 39, F-75005 Paris, France

^5^ Département de Botanique, Faculté des Sciences Pharmaceutiques et Biologiques, Université Lille - Nord de France B. P. 83, 59006 Lille Cedex, France

* Corresponding author: adrien.taudiere@cefe.cnrs.fr Tel: +33 (0)4.67.61.32.62 Fax: +33 (0)4.67.61.41.21.38

Table of contents

[1. Supplementary Data 3](#_Toc425258809)

[Supplementary Table S1: Binary matrix of association between 16 plant species and 411 ECM fungal species in Corsica. Value 1 at position (*p*, *f*) indicates that the fungal species *f* has been reported to be an ECM symbiont of the plant species *p*. 3](#_Toc425258810)

[2. Supplementary References 28](#_Toc425258811)

[Supplementary References S2: References used to build the dataset. 28](#_Toc425258812)

[3. Supplementary Methods 33](#_Toc425258813)

[Supplementary Methods S3: Species included in the analysis (see also Fig. S4) 33](#_Toc425258814)

[4. Supplementary Figures 35](#_Toc425258815)

[Figure S4: Workflow of data filtering to build the dataset 35](#_Toc425258816)

[Figure S5: Equivalent of figure 3 in a fungal perspective 36](#_Toc425258817)

[Figure S6: Distributions of plants’ networks metrics 37](#_Toc425258818)

[Figure S7: Matrix representation of the plant projected network 38](#_Toc425258819)

[Figure S8: Enhanced plants’ networks metrics comparisons between early stage and late stage plants species 39](#_Toc425258820)

[Figure S9: Average distance between fungal communities regardless their host ecology. 40](#_Toc425258821)

[Figure S10: Plant projected weighted network. 41](#_Toc425258822)

[5. Supplementary Script 42](#_Toc425258823)

[Supplementary Material S11: R function to calculate networks metrics used in this paper 42](#_Toc425258824)

## Supplementary Data

Supplementary Table S1: **Binary matrix of association between 16 plant species and 411 ECM fungal species in Corsica. Value 1 at position (*p*, *f*) indicates that the fungal species *f* has been reported to be an ECM symbiont of the plant species *p*. The following abbreviations were used to indicate plant genera: Ab.: Abies; Al.: Alnus; B.: Betula; Ca.: Castanea; Co.: Corylus; F.: Fagus; H.: Halimium; Pi.: Pinus; Po.: Populus; Q.: Quercus.**

|  | *Cistus spp* | *H. halimifolium* | *Ca. sativa* | *F. sylvatica* | *Q. ilex* | *Q. suber* | *Ab. alba* | *Pi. nigra* | *Pi. pinaster* | *Salix spp* | *Al. alnobetula* | *Al. cordata* | *Al. glutinosa* | *B. pendula* | *Co. avellana* | *Po. alba* |
| --- | --- | --- | --- | --- | --- | --- | --- | --- | --- | --- | --- | --- | --- | --- | --- | --- |
| **Plants full names** | *Cistus spp* | *Halimium halimifolium* | *Castanea sativa* | *Fagus sylvatica* | *Quercus ilex* | *Quercus suber* | *Abies alba* | *Pinus nigra subsp. laricio* | *Pinus pinaster* | *Salix spp* | *Alnus alnobetula subsp. suaveolens* | *Alnus cordata* | *Alnus glutinosa* | *Betula pendula* | *Corylus avellana* | *Populus alba* |
| **Fungus names** |  |  |  |  |  |  |  |  |  |  |  |  |  |  |  |  |
| **Alnicola badia** Kühner | 0 | 0 | 0 | 0 | 0 | 0 | 0 | 0 | 0 | 0 | 1 | 0 | 0 | 0 | 0 | 0 |
| **Alnicola badiofusca** P.-A. Moreau | 0 | 0 | 0 | 0 | 0 | 0 | 0 | 0 | 0 | 0 | 1 | 0 | 0 | 0 | 0 | 0 |
| **Alnicola citrinella** P.-A. Moreau & A. De Haan | 0 | 0 | 0 | 0 | 0 | 0 | 0 | 0 | 0 | 0 | 0 | 0 | 1 | 0 | 0 | 0 |
| **Alnicola escharoides** (Fr.: Fr.) Romagn. | 0 | 0 | 0 | 0 | 0 | 0 | 0 | 0 | 0 | 0 | 1 | 1 | 1 | 0 | 0 | 0 |
| **Alnicola inculta** (Peck) Singer | 0 | 0 | 0 | 0 | 0 | 0 | 0 | 0 | 0 | 0 | 1 | 0 | 1 | 0 | 0 | 0 |
| **Alnicola longicystis** P.-A. Moreau *et al.* | 0 | 0 | 0 | 0 | 0 | 0 | 0 | 0 | 0 | 0 | 0 | 0 | 1 | 0 | 0 | 0 |
| **Alnicola macrospora** J. Favre | 0 | 0 | 0 | 0 | 0 | 0 | 0 | 0 | 0 | 1 | 0 | 0 | 0 | 0 | 0 | 0 |
| **Alnicola pallidifolia** P.-A. Moreau & Peintner | 0 | 0 | 0 | 0 | 0 | 0 | 0 | 0 | 0 | 0 | 1 | 0 | 0 | 0 | 0 | 0 |
| **Alnicola salabertii** P.-A. Moreau & Guy Garcia | 0 | 0 | 0 | 0 | 0 | 0 | 0 | 0 | 0 | 0 | 0 | 1 | 0 | 0 | 0 | 0 |
| **Alnicola subconspersa** (P.D. Orton) Bon | 0 | 0 | 0 | 0 | 0 | 0 | 0 | 0 | 0 | 0 | 0 | 1 | 1 | 0 | 0 | 0 |
| **Alnicola submelinoides** Kühner | 0 | 0 | 0 | 0 | 0 | 0 | 0 | 0 | 0 | 0 | 1 | 0 | 0 | 0 | 0 | 0 |
| **Alnicola umbrina (**R.Maire) Kühner | 0 | 0 | 0 | 0 | 0 | 0 | 0 | 0 | 0 | 0 | 0 | 0 | 1 | 0 | 0 | 0 |
| **Alpova alpestris** P.-A. Moreau & F. Richard | 0 | 0 | 0 | 0 | 0 | 0 | 0 | 0 | 0 | 0 | 1 | 0 | 0 | 0 | 0 | 0 |
| **Alpova corsicus** P.-A. Moreau & F. Richard | 0 | 0 | 0 | 0 | 0 | 0 | 0 | 0 | 0 | 0 | 1 | 1 | 1 | 0 | 0 | 0 |
| **Amanita boudieri** Barla | 1 | 0 | 0 | 0 | 1 | 0 | 0 | 0 | 1 | 0 | 0 | 0 | 0 | 0 | 0 | 0 |
| **Amanita caesarea** (Scop. : Fr.) Pers. | 0 | 0 | 1 | 0 | 1 | 1 | 0 | 0 | 0 | 0 | 0 | 0 | 0 | 0 | 0 | 0 |
| **Amanita cistetorum** Pacioni & Contu | 1 | 1 | 0 | 0 | 0 | 0 | 0 | 0 | 0 | 0 | 0 | 0 | 0 | 0 | 0 | 0 |
| **Amanita citrina** (Schaeff.: Fr.) Pers. | 0 | 0 | 1 | 1 | 1 | 1 | 1 | 1 | 1 | 0 | 0 | 0 | 0 | 1 | 0 | 0 |
| **Amanita crocea** (Quél.) Singer | 0 | 0 | 0 | 1 | 1 | 1 | 0 | 0 | 0 | 0 | 0 | 0 | 0 | 0 | 1 | 0 |
| **Amanita curtipes** E.-J. Gilbert | 1 | 1 | 0 | 0 | 1 | 0 | 0 | 0 | 1 | 0 | 0 | 0 | 0 | 0 | 0 | 0 |
| **Amanita excelsa** (Fr.: Fr.) Bertill. | 0 | 0 | 1 | 1 | 0 | 0 | 1 | 1 | 0 | 0 | 0 | 0 | 0 | 1 | 1 | 0 |
| **Amanita flavescens** (E.-J. Gilbert & S. Lundell) Contu | 0 | 0 | 0 | 0 | 1 | 1 | 0 | 0 | 0 | 0 | 0 | 0 | 0 | 0 | 0 | 0 |
| **Amanita franchetii var. queletii** (Bon & Dennis) Neville & Poumarat | 0 | 0 | 0 | 1 | 1 | 0 | 0 | 0 | 0 | 0 | 0 | 0 | 0 | 0 | 0 | 0 |
| **Amanita friabilis** (P.Karst.) Bas | 0 | 0 | 0 | 0 | 0 | 0 | 0 | 0 | 0 | 0 | 1 | 0 | 1 | 0 | 0 | 0 |
| **Amanita fulva** (Schaeff.: Fr.) Fr. | 0 | 0 | 0 | 0 | 0 | 0 | 0 | 0 | 0 | 0 | 0 | 0 | 0 | 1 | 0 | 0 |
| **Amanita junquillea** Quél. | 0 | 0 | 0 | 0 | 1 | 1 | 0 | 0 | 0 | 0 | 0 | 0 | 0 | 0 | 0 | 0 |
| **Amanita lividopallescens** (Boud.) Kühner & Romagn. | 0 | 0 | 0 | 0 | 1 | 1 | 0 | 0 | 0 | 0 | 0 | 0 | 0 | 0 | 0 | 0 |
| **Amanita lividopallescens_var.tigrina** Bon | 0 | 0 | 0 | 0 | 1 | 1 | 0 | 0 | 0 | 0 | 0 | 0 | 0 | 0 | 0 | 0 |
| **Amanita mairei** Foley | 0 | 0 | 0 | 0 | 0 | 0 | 0 | 1 | 1 | 0 | 0 | 0 | 0 | 0 | 0 | 0 |
| **Amanita malleata Piane [nomen dubium]** | 1 | 1 | 0 | 0 | 1 | 1 | 0 | 0 | 0 | 0 | 0 | 0 | 0 | 0 | 0 | 0 |
| **Amanita muscaria** (L.: Fr.) Lam. | 0 | 0 | 0 | 1 | 1 | 1 | 1 | 1 | 0 | 0 | 0 | 0 | 0 | 0 | 0 | 0 |
| **Amanita muscaria_**var.*inzengae* Neville & Poumarat | 1 | 1 | 0 | 0 | 0 | 0 | 0 | 0 | 0 | 0 | 0 | 0 | 0 | 0 | 0 | 0 |
| **Amanita ovoidea** (Bull.: Fr.) Link | 0 | 0 | 0 | 0 | 1 | 1 | 0 | 0 | 0 | 0 | 0 | 0 | 0 | 0 | 0 | 0 |
| **Amanita pantherina** (DC: Fr.) Krombh. | 1 | 1 | 1 | 1 | 1 | 1 | 1 | 1 | 1 | 0 | 1 | 1 | 1 | 0 | 0 | 0 |
| **Amanita pantherina_**f.*abietum* (E.-J. Gilbert) Neville & Poumarat | 0 | 0 | 0 | 0 | 0 | 0 | 1 | 0 | 0 | 0 | 0 | 0 | 0 | 0 | 0 | 0 |
| **Amanita phalloides** (Vaill.: Fr.) Link | 0 | 0 | 1 | 1 | 1 | 1 | 0 | 0 | 0 | 0 | 0 | 0 | 0 | 0 | 1 | 0 |
| **Amanita rubescens** (Pers.: Fr.) Gray | 0 | 0 | 1 | 1 | 1 | 1 | 1 | 1 | 1 | 0 | 0 | 0 | 1 | 1 | 1 | 0 |
| **Amanita separata** Contu | 0 | 0 | 0 | 0 | 1 | 1 | 0 | 0 | 0 | 0 | 0 | 0 | 0 | 0 | 0 | 0 |
|  |  |  |  |  |  |  |  |  |  |  |  |  |  |  |  |  |
| **Amanita simulans** Contu | 0 | 0 | 0 | 0 | 0 | 0 | 0 | 0 | 0 | 1 | 0 | 0 | 0 | 0 | 0 | 1 |
| **Amanita spissa** (Fr.) Kummer | 0 | 0 | 1 | 1 | 1 | 1 | 0 | 0 | 0 | 0 | 0 | 0 | 0 | 0 | 1 | 0 |
| **Amanita submembranacea** (Bon) Gröger | 0 | 0 | 0 | 1 | 0 | 0 | 0 | 0 | 0 | 0 | 0 | 0 | 0 | 0 | 0 | 0 |
| **Amanita umbrinolutea** (Gillet) Bat. | 0 | 0 | 0 | 1 | 0 | 0 | 0 | 0 | 0 | 0 | 0 | 0 | 0 | 0 | 0 | 0 |
| **Amanita vaginata f. alba** (Bull.) Veselý | 1 | 1 | 1 | 1 | 1 | 1 | 1 | 1 | 1 | 1 | 0 | 0 | 0 | 1 | 1 | 0 |
| **Amanita verna** (Bull.: Fr.) Lam. | 0 | 0 | 0 | 0 | 1 | 1 | 0 | 0 | 0 | 0 | 0 | 0 | 0 | 0 | 0 | 0 |
| **Amanita virosa** Lam. | 0 | 0 | 1 | 0 | 1 | 1 | 0 | 0 | 0 | 0 | 0 | 0 | 0 | 0 | 0 | 0 |
| **Aureoboletus gentilis** (Quél.) Pouzar | 0 | 0 | 0 | 0 | 1 | 1 | 0 | 0 | 0 | 0 | 0 | 0 | 0 | 0 | 0 | 0 |
| **Bankera fuligineoalba** (Schmidt : Fr.) Pouz. | 0 | 0 | 0 | 0 | 0 | 0 | 0 | 1 | 1 | 0 | 0 | 0 | 0 | 0 | 0 | 0 |
| **Boletopsis subsquamosa** (L.: Fr.) Kotl. & Pouzar | 0 | 0 | 0 | 0 | 0 | 0 | 0 | 0 | 1 | 0 | 0 | 0 | 0 | 0 | 0 | 0 |
| **Boletus aereus** Bull. : Fr. | 1 | 1 | 0 | 0 | 1 | 1 | 0 | 0 | 0 | 0 | 0 | 0 | 0 | 0 | 0 | 0 |
| **Boletus aestivalis** (Paulet) Fr. | 0 | 0 | 0 | 1 | 0 | 0 | 0 | 0 | 0 | 0 | 0 | 0 | 0 | 0 | 0 | 0 |
| **Boletus aff. flavosanguineus** Simonini & Lavorato | 0 | 0 | 0 | 0 | 1 | 1 | 0 | 0 | 0 | 0 | 0 | 0 | 0 | 0 | 0 | 0 |
| **Boletus appendiculatus** Schaeff. : Fr. | 0 | 0 | 0 | 0 | 1 | 1 | 0 | 0 | 0 | 0 | 0 | 0 | 0 | 0 | 0 | 0 |
| **Boletus calopus** Pers. : Fr. | 0 | 0 | 0 | 1 | 1 | 1 | 0 | 0 | 0 | 0 | 0 | 0 | 0 | 0 | 0 | 0 |
| **Boletus comptus** Simonini | 0 | 0 | 0 | 1 | 0 | 0 | 0 | 0 | 0 | 0 | 0 | 0 | 0 | 0 | 0 | 0 |
| **Boletus edulis** Bull. : Fr. | 0 | 0 | 1 | 1 | 1 | 1 | 1 | 1 | 1 | 0 | 0 | 0 | 0 | 1 | 0 | 0 |
| **Boletus erythropus** Pers. : Fr. | 1 | 0 | 1 | 1 | 1 | 1 | 1 | 1 | 1 | 0 | 0 | 0 | 0 | 0 | 0 | 0 |
| **Boletus fragrans** Vitt. | 0 | 0 | 0 | 0 | 1 | 1 | 0 | 0 | 0 | 0 | 0 | 0 | 0 | 0 | 0 | 0 |
| **Boletus impolitus** Fr. | 0 | 0 | 0 | 0 | 1 | 1 | 0 | 0 | 0 | 0 | 0 | 0 | 0 | 0 | 0 | 0 |
| **Boletus legaliae** Pilàt | 0 | 0 | 0 | 0 | 1 | 1 | 0 | 0 | 0 | 0 | 0 | 0 | 0 | 0 | 0 | 0 |
| **Boletus lupinus** Fr. | 0 | 0 | 0 | 0 | 1 | 1 | 0 | 0 | 0 | 0 | 0 | 0 | 0 | 0 | 0 | 0 |
| **Boletus luridus** Schaeff.: Fr. | 0 | 0 | 1 | 1 | 1 | 1 | 1 | 1 | 1 | 0 | 0 | 0 | 0 | 0 | 0 | 0 |
| **Boletus permagnificus** Pöder | 0 | 0 | 0 | 0 | 1 | 1 | 0 | 0 | 0 | 0 | 0 | 0 | 0 | 0 | 0 | 0 |
| **Boletus pinophilus** Pilát & Dermek | 0 | 0 | 0 | 0 | 0 | 0 | 0 | 1 | 1 | 0 | 0 | 0 | 0 | 0 | 0 | 0 |
| **Boletus pulverulentus** Opat. | 0 | 0 | 0 | 0 | 0 | 1 | 0 | 0 | 0 | 0 | 0 | 0 | 0 | 0 | 1 | 0 |
| **Boletus queletii** Schulz. | 0 | 0 | 0 | 0 | 1 | 1 | 0 | 0 | 0 | 0 | 0 | 0 | 0 | 0 | 0 | 0 |
| **Boletus queletii_var.discolor** | 0 | 0 | 0 | 0 | 1 | 1 | 0 | 0 | 0 | 0 | 0 | 0 | 0 | 0 | 0 | 0 |
| **Boletus radicans** Pers. : Fr. | 0 | 0 | 0 | 0 | 1 | 1 | 0 | 0 | 0 | 0 | 0 | 0 | 0 | 0 | 0 | 0 |
| **Boletus regius** Krombh. | 0 | 0 | 0 | 0 | 1 | 1 | 0 | 0 | 0 | 0 | 0 | 0 | 0 | 0 | 0 | 0 |
| **Boletus rhodoxanthus** (Krombh.) Kallenb. | 0 | 0 | 0 | 0 | 1 | 1 | 0 | 0 | 0 | 0 | 0 | 0 | 0 | 0 | 0 | 0 |
|  |  |  |  |  |  |  |  |  |  |  |  |  |  |  |  |  |
| **Boletus satanas** Lenz | 0 | 0 | 0 | 1 | 1 | 0 | 0 | 0 | 0 | 0 | 0 | 0 | 0 | 0 | 0 | 0 |
| **Boletus spretus** Bertéa | 0 | 0 | 0 | 0 | 1 | 1 | 0 | 0 | 0 | 0 | 0 | 0 | 0 | 0 | 0 | 0 |
| **Cantharellus ferruginascens** P.D. Orton | 0 | 0 | 0 | 0 | 1 | 0 | 0 | 0 | 0 | 0 | 0 | 0 | 0 | 0 | 0 | 0 |
| **Cantharellus lilacinopruinatus** Hermitte, Eyssartier & Poumarat | 0 | 0 | 0 | 0 | 1 | 0 | 0 | 0 | 0 | 0 | 0 | 0 | 0 | 0 | 0 | 0 |
| **Cantharellus pallens** (=**C. subpruinosus**) | 0 | 0 | 1 | 1 | 1 | 1 | 1 | 1 | 1 | 0 | 0 | 0 | 0 | 1 | 1 | 0 |
| **Catathelasma imperiale** (Fr.) Singer | 0 | 0 | 0 | 0 | 0 | 0 | 1 | 0 | 0 | 0 | 0 | 0 | 0 | 0 | 0 | 0 |
| **Chalciporus piperatus** (Bull. : Fr.) Bat. | 0 | 0 | 0 | 0 | 0 | 0 | 1 | 0 | 0 | 0 | 0 | 0 | 0 | 0 | 0 | 0 |
| **Chroogomphus corallinus** O.K. Miller & Watling | 0 | 0 | 0 | 0 | 0 | 0 | 0 | 1 | 1 | 0 | 0 | 0 | 0 | 0 | 0 | 0 |
| **Chroogomphus rutilus** (Schaeff. : Fr.) O.K. Miller | 0 | 0 | 0 | 0 | 0 | 0 | 0 | 1 | 1 | 0 | 0 | 0 | 0 | 0 | 0 | 0 |
| **Clavariadelphus pistillaris** (L.: Fr.) Donk | 0 | 0 | 0 | 1 | 1 | 0 | 0 | 0 | 0 | 0 | 0 | 0 | 0 | 0 | 0 | 0 |
| **Clavulina cinerea** (Bull.: Fr.) J. Schröt. | 0 | 0 | 1 | 1 | 1 | 1 | 1 | 1 | 1 | 1 | 0 | 0 | 0 | 1 | 1 | 1 |
| **Clavulina coralloides** (L.: Fr.) J. Schröt. | 1 | 0 | 1 | 1 | 1 | 1 | 1 | 1 | 1 | 1 | 0 | 0 | 0 | 1 | 1 | 1 |
| **Clitopilus cystidiatus** Hauskn**.** & Noordel. | 0 | 0 | 0 | 1 | 1 | 1 | 0 | 0 | 0 | 0 | 0 | 0 | 0 | 0 | 1 | 0 |
| **Coltricia perennis** (L. : Fr.) Murrill | 1 | 1 | 1 | 1 | 1 | 1 | 0 | 1 | 1 | 0 | 1 | 1 | 1 | 0 | 0 | 0 |
| **Cortinarius alnetorum** Velen. | 0 | 0 | 0 | 0 | 0 | 0 | 0 | 0 | 0 | 0 | 1 | 1 | 1 | 0 | 0 | 0 |
| **Cortinarius atropusillus** | 0 | 0 | 0 | 0 | 0 | 0 | 0 | 0 | 0 | 0 | 1 | 1 | 1 | 0 | 0 | 0 |
| **Cortinarius badiovestitus** | 0 | 0 | 0 | 0 | 0 | 0 | 0 | 0 | 0 | 0 | 1 | 0 | 0 | 0 | 0 | 0 |
| **Cortinarius cinnabarinus** Fr**.** | 0 | 0 | 0 | 1 | 0 | 0 | 0 | 0 | 0 | 0 | 0 | 0 | 0 | 0 | 0 | 0 |
| **Cortinarius cinnamomeoluteus** P.D. Orton | 0 | 0 | 0 | 0 | 0 | 0 | 0 | 0 | 0 | 1 | 0 | 0 | 0 | 0 | 0 | 0 |
| **Cortinarius cohabitans** P.Karst**.** | 0 | 0 | 0 | 0 | 0 | 0 | 0 | 0 | 0 | 1 | 0 | 0 | 0 | 0 | 0 | 0 |
| **Cortinarius diosmus** var. **araneosovolvatus** | 0 | 0 | 0 | 0 | 1 | 0 | 0 | 0 | 0 | 0 | 0 | 0 | 0 | 0 | 0 | 0 |
| **Cortinarius elatior** var. **ochraceoplicatus** | 0 | 0 | 1 | 1 | 1 | 1 | 0 | 0 | 0 | 0 | 0 | 0 | 0 | 0 | 0 | 0 |
| **Cortinarius elatior** Fr. | 0 | 0 | 1 | 1 | 1 | 1 | 0 | 0 | 0 | 0 | 0 | 0 | 0 | 0 | 0 | 0 |
| **Cortinarius elegantior** (Fr.) Fr. | 0 | 0 | 0 | 0 | 0 | 0 | 1 | 0 | 0 | 0 | 0 | 0 | 0 | 0 | 0 | 0 |
| **Cortinarius helvelloides** Fr.: Fr. | 0 | 0 | 0 | 0 | 0 | 0 | 0 | 0 | 0 | 0 | 1 | 1 | 1 | 0 | 0 | 0 |
| **Cortinarius humicola** (Quél.) Maire | 0 | 0 | 0 | 1 | 0 | 0 | 0 | 0 | 0 | 0 | 0 | 0 | 0 | 0 | 0 | 0 |
| **Cortinarius pulchellus** | 0 | 0 | 0 | 0 | 0 | 0 | 0 | 0 | 0 | 0 | 1 | 1 | 1 | 0 | 0 | 0 |
| **Cortinarius splendens** R. Henry | 0 | 0 | 0 | 1 | 0 | 0 | 0 | 0 | 0 | 0 | 0 | 0 | 0 | 0 | 0 | 0 |
| **Cortinarius triformis** Fr. | 0 | 0 | 0 | 1 | 0 | 0 | 0 | 0 | 0 | 0 | 0 | 0 | 0 | 0 | 0 | 0 |
| **Craterellus lutescens** (Pers. : Fr.) Fr. | 0 | 0 | 0 | 0 | 0 | 0 | 0 | 1 | 1 | 0 | 0 | 0 | 0 | 0 | 0 | 0 |
| **Craterellus tubaeformis** (Bull. : Fr.) Quél. | 0 | 0 | 1 | 1 | 1 | 1 | 1 | 1 | 1 | 0 | 0 | 0 | 0 | 1 | 0 | 0 |
| **Entoloma lividoalbum** (Kühner & Romagn.) Kubicka | 0 | 0 | 0 | 0 | 1 | 1 | 0 | 0 | 0 | 0 | 0 | 0 | 0 | 0 | 0 | 0 |
| **Entoloma lividum** Bull.Quél. | 0 | 0 | 0 | 0 | 1 | 0 | 0 | 0 | 0 | 0 | 0 | 0 | 0 | 0 | 0 | 0 |
| **Entoloma rhodopolium** (Fr.: Fr.) Kummer | 0 | 0 | 0 | 1 | 1 | 1 | 0 | 0 | 0 | 0 | 0 | 0 | 0 | 1 | 1 | 1 |
| **Geopyxis carbonaria** | 0 | 0 | 0 | 0 | 0 | 0 | 0 | 1 | 1 | 0 | 0 | 0 | 0 | 0 | 0 | 0 |
| **Gomphidius glutinosus** (Schaeff. : Fr.) Fr. | 0 | 0 | 0 | 0 | 0 | 0 | 1 | 0 | 0 | 0 | 0 | 0 | 0 | 0 | 0 | 0 |
| **Gomphidius roseus** (Nees : Fr.) Gillet | 0 | 0 | 0 | 0 | 0 | 0 | 0 | 1 | 1 | 0 | 0 | 0 | 0 | 0 | 0 | 0 |
| **Gomphus clavatus** (Pers.: Fr.) Gray | 0 | 0 | 0 | 0 | 0 | 0 | 1 | 0 | 0 | 0 | 0 | 0 | 0 | 0 | 0 | 0 |
| **Gyrodon lividus** (Bull.: Fr.) P. Karst. | 0 | 0 | 0 | 0 | 0 | 0 | 0 | 0 | 0 | 0 | 0 | 0 | 1 | 0 | 0 | 0 |
| **Gyromitra esculenta** (Pers.: Fr.) Fr. | 0 | 0 | 0 | 0 | 0 | 0 | 0 | 1 | 1 | 0 | 0 | 0 | 0 | 0 | 0 | 0 |
| **Gyromitra infula** (Schaeff.: Fr.) Quél. | 0 | 0 | 0 | 0 | 0 | 0 | 0 | 1 | 1 | 0 | 0 | 0 | 0 | 0 | 0 | 0 |
| **Gyroporus castaneus** (Bull. : Fr.) Quél. | 0 | 0 | 0 | 0 | 1 | 1 | 0 | 0 | 0 | 0 | 0 | 0 | 0 | 0 | 0 | 0 |
| **Gyroporus cyanescens** (Bull. : Fr.) Quél. | 0 | 0 | 1 | 0 | 1 | 1 | 0 | 0 | 0 | 0 | 0 | 0 | 0 | 0 | 0 | 0 |
|  |  |  |  |  |  |  |  |  |  |  |  |  |  |  |  |  |
| **Hebeloma cistophilum** Maire | 1 | 1 | 0 | 0 | 0 | 0 | 0 | 0 | 0 | 0 | 0 | 0 | 0 | 0 | 0 | 0 |
| **Hebeloma erumpens** Contu | 1 | 0 | 0 | 0 | 0 | 0 | 0 | 0 | 0 | 0 | 0 | 0 | 0 | 0 | 0 | 0 |
| **Hebeloma laterinum** (Batsch) Vesterh. | 0 | 0 | 0 | 0 | 1 | 0 | 0 | 0 | 1 | 0 | 0 | 0 | 0 | 0 | 0 | 0 |
| **Hebeloma lutense** Romagn. | 0 | 0 | 0 | 0 | 0 | 0 | 0 | 0 | 0 | 1 | 0 | 0 | 0 | 0 | 0 | 0 |
| **Hebeloma porphyrosporum** | 0 | 0 | 0 | 0 | 1 | 0 | 0 | 0 | 1 | 0 | 0 | 0 | 0 | 0 | 0 | 0 |
| **Hebeloma sinapizans** (Paulet) Gillet | 0 | 0 | 0 | 0 | 1 | 0 | 0 | 0 | 0 | 0 | 0 | 0 | 0 | 0 | 0 | 0 |
| **Hebeloma theobrominum** Quadr. | 0 | 0 | 0 | 0 | 1 | 1 | 1 | 0 | 0 | 0 | 0 | 0 | 0 | 0 | 0 | 0 |
| **Hebeloma velutipes** Bruchet | 0 | 0 | 1 | 1 | 0 | 0 | 1 | 1 | 1 | 1 | 0 | 0 | 0 | 1 | 1 | 1 |
| **Helvella crispa** Scop.: Fr. | 0 | 0 | 0 | 0 | 1 | 1 | 0 | 0 | 0 | 0 | 0 | 0 | 0 | 0 | 0 | 0 |
| **Helvella elastica** Bull.: Fr. | 0 | 0 | 0 | 0 | 1 | 0 | 0 | 0 | 0 | 0 | 0 | 0 | 0 | 0 | 0 | 0 |
| **Helvella juniperina** M. Filippa & Baiano | 0 | 0 | 0 | 0 | 1 | 0 | 0 | 0 | 0 | 0 | 0 | 0 | 0 | 0 | 0 | 0 |
| **Humaria hemisphaerica** (Wigg.: Fr.) Fuckel | 0 | 0 | 1 | 1 | 1 | 1 | 1 | 1 | 1 | 1 | 0 | 0 | 0 | 1 | 1 | 1 |
| **Hydnellum aurantiacum** (Batsch : Fr.) P. Karst. | 0 | 0 | 0 | 0 | 0 | 0 | 0 | 0 | 1 | 0 | 0 | 0 | 0 | 0 | 0 | 0 |
| **Hydnellum caeruleum** (Hornem.: Fr.) P. Karst. | 0 | 0 | 0 | 0 | 0 | 0 | 1 | 1 | 1 | 0 | 0 | 0 | 0 | 0 | 0 | 0 |
|  |  |  |  |  |  |  |  |  |  |  |  |  |  |  |  |  |
| **Hydnellum concrescens** (Pers. : Schw.) Banker | 0 | 0 | 0 | 0 | 1 | 0 | 0 | 0 | 0 | 0 | 0 | 0 | 0 | 0 | 0 | 0 |
| **Hydnellum ferrugineum** (Fr.: Fr.) P. Karst. | 0 | 0 | 1 | 0 | 1 | 1 | 1 | 0 | 0 | 0 | 0 | 0 | 0 | 0 | 0 | 0 |
| **Hydnellum scrobiculatum** (Fr.: Fr.) P. Karst. | 0 | 0 | 0 | 0 | 1 | 0 | 0 | 0 | 0 | 0 | 0 | 0 | 0 | 0 | 0 | 0 |
| **Hydnellum spongiosipes** (Peck) Pouzar | 0 | 0 | 1 | 0 | 1 | 1 | 0 | 0 | 0 | 0 | 0 | 0 | 0 | 0 | 0 | 0 |
| **Hydnocystis piligera** Tul. & C. Tul. | 0 | 0 | 0 | 0 | 1 | 0 | 0 | 0 | 0 | 0 | 0 | 0 | 0 | 0 | 0 | 0 |
| **Hydnum albidum** Peck | 0 | 0 | 0 | 1 | 0 | 0 | 0 | 1 | 1 | 0 | 0 | 0 | 0 | 0 | 0 | 0 |
| **Hydnum repandum** L. : Fr. | 0 | 0 | 1 | 1 | 1 | 1 | 1 | 1 | 1 | 0 | 0 | 0 | 0 | 0 | 0 | 0 |
| **Hydnum rufescens** Schaeff. : Fr. | 0 | 0 | 1 | 1 | 1 | 1 | 0 | 0 | 0 | 0 | 0 | 0 | 0 | 0 | 0 | 0 |
| **Hygrophorus agathosmus** (Fr.) Fr**.** | 0 | 0 | 0 | 0 | 0 | 0 | 0 | 0 | 1 | 0 | 0 | 0 | 0 | 0 | 0 | 0 |
| **Hygrophorus calophyllus** P. Karst. | 0 | 0 | 0 | 0 | 0 | 0 | 0 | 0 | 1 | 0 | 0 | 0 | 0 | 0 | 0 | 0 |
| **Hygrophorus camarophyllus** (Alb. & Schwein. : Fr.) Dumée, Grandjean & R. Maire | 0 | 0 | 0 | 0 | 0 | 0 | 1 | 0 | 1 | 0 | 0 | 0 | 0 | 0 | 0 | 0 |
| **Hygrophorus cossus** (Sow.) Fr. | 0 | 0 | 0 | 0 | 1 | 1 | 0 | 0 | 0 | 0 | 0 | 0 | 0 | 0 | 0 | 0 |
| **Hygrophorus eburneus** (Bull. : Fr.) Fr. | 0 | 0 | 0 | 1 | 0 | 0 | 0 | 0 | 0 | 0 | 0 | 0 | 0 | 0 | 0 | 0 |
| **Hygrophorus gliocyclus** Fr. | 0 | 0 | 0 | 0 | 0 | 0 | 0 | 0 | 1 | 0 | 0 | 0 | 0 | 0 | 0 | 0 |
| **Hygrophorus hypothejus** (Fr.: Fr.) Fr. | 0 | 0 | 0 | 0 | 0 | 0 | 0 | 1 | 1 | 0 | 0 | 0 | 0 | 0 | 0 | 0 |
| **Hygrophorus latitabundus** Britzelm. | 0 | 0 | 0 | 0 | 0 | 0 | 0 | 0 | 1 | 0 | 0 | 0 | 0 | 0 | 0 | 0 |
| **Hygrophorus leucophaeus** (Scop.) Fr. | 0 | 0 | 0 | 1 | 0 | 0 | 0 | 0 | 0 | 0 | 0 | 0 | 0 | 0 | 0 | 0 |
| **Hygrophorus marzuolus** (Fr.: Fr.) Bres. | 0 | 0 | 0 | 1 | 0 | 0 | 1 | 0 | 1 | 0 | 0 | 0 | 0 | 0 | 0 | 0 |
| **Hygrophorus mesotephrus** Berk. & Broome | 0 | 0 | 0 | 1 | 0 | 0 | 0 | 0 | 0 | 0 | 0 | 0 | 0 | 0 | 0 | 0 |
| **Hygrophorus nemoreus** (Pers.: Fr.) Fr. | 0 | 0 | 0 | 0 | 1 | 1 | 0 | 0 | 0 | 0 | 0 | 0 | 0 | 0 | 0 | 0 |
| **Hygrophorus penarioides** Jacobss. & E. Larss. | 0 | 0 | 0 | 0 | 1 | 1 | 0 | 0 | 0 | 0 | 0 | 0 | 0 | 0 | 0 | 0 |
| **Hygrophorus persoonii** Arnolds | 0 | 0 | 0 | 0 | 1 | 1 | 0 | 0 | 0 | 0 | 0 | 0 | 0 | 0 | 0 | 0 |
| **Hygrophorus poetarum** R. Heim | 0 | 0 | 0 | 1 | 0 | 0 | 0 | 0 | 0 | 0 | 0 | 0 | 0 | 0 | 0 | 0 |
| **Hygrophorus pseudodiscoideus** var. **cistophilus** Bon & G. Riousset | 1 | 0 | 0 | 0 | 0 | 0 | 0 | 0 | 0 | 0 | 0 | 0 | 0 | 0 | 0 | 0 |
| **Hygrophorus pudorinus** (Fr.: Fr.) Fr. | 0 | 0 | 0 | 0 | 0 | 0 | 1 | 0 | 0 | 0 | 0 | 0 | 0 | 0 | 0 | 0 |
| **Hygrophorus russula** (Schaeff.: Fr.) Quél. | 0 | 0 | 0 | 0 | 1 | 1 | 0 | 0 | 0 | 0 | 0 | 0 | 0 | 0 | 0 | 0 |
| **Inocybe adaequata** (=**I. jurana**) | 0 | 0 | 0 | 0 | 1 | 0 | 0 | 0 | 0 | 0 | 0 | 0 | 0 | 0 | 0 | 0 |
| **Inocybe aff. xanthomelaena** Kühner & Boursier | 0 | 0 | 0 | 0 | 0 | 0 | 0 | 0 | 0 | 0 | 1 | 0 | 0 | 0 | 0 | 0 |
| **Inocybe alnea** | 0 | 0 | 0 | 0 | 0 | 0 | 0 | 0 | 0 | 0 | 1 | 0 | 1 | 0 | 0 | 0 |
| **Inocybe calospora** Quél. | 0 | 0 | 0 | 0 | 1 | 0 | 0 | 0 | 0 | 0 | 0 | 0 | 1 | 1 | 0 | 0 |
| **Inocybe cerina** (Malençon) Bon | 0 | 0 | 0 | 0 | 1 | 1 | 0 | 0 | 0 | 0 | 0 | 0 | 0 | 0 | 0 | 0 |
| **Inocybe cervicolor** (Pers.) Quél. | 0 | 0 | 0 | 0 | 0 | 0 | 1 | 0 | 0 | 0 | 0 | 0 | 0 | 0 | 0 | 0 |
| **Inocybe cf straminipes** | 0 | 0 | 0 | 0 | 0 | 0 | 0 | 0 | 0 | 1 | 0 | 0 | 0 | 0 | 0 | 0 |
| **Inocybe cookei** Quél. | 0 | 0 | 0 | 0 | 1 | 1 | 0 | 0 | 0 | 0 | 0 | 0 | 0 | 0 | 0 | 0 |
| **Inocybe crucifera** | 0 | 0 | 0 | 0 | 0 | 0 | 0 | 0 | 0 | 0 | 1 | 0 | 0 | 0 | 0 | 0 |
| **Inocybe curvipes** | 0 | 0 | 1 | 1 | 1 | 1 | 0 | 1 | 1 | 0 | 0 | 0 | 0 | 1 | 1 | 1 |
| **Inocybe** decemgibbosa (Kühner & Boursier) Vauras | 0 | 0 | 0 | 0 | 1 | 0 | 0 | 0 | 0 | 0 | 0 | 0 | 0 | 0 | 0 | 0 |
| **Inocybe eutheles** Berk. & Broome (ss. Singer) | 0 | 0 | 0 | 0 | 0 | 0 | 1 | 1 | 1 | 0 | 0 | 0 | 0 | 0 | 0 | 0 |
| **Inocybe fibrosoides** Kühner & Boursier | 0 | 0 | 0 | 0 | 0 | 1 | 0 | 0 | 0 | 0 | 0 | 0 | 0 | 0 | 0 | 0 |
| **Inocybe geophylla** (Sow.: Fr.) Kummer | 0 | 0 | 1 | 1 | 1 | 1 | 1 | 1 | 1 | 1 | 0 | 0 | 0 | 1 | 1 | 1 |
| **Inocybe geophylla_var.lilacina (**Peck) Gillet | 0 | 0 | 0 | 0 | 1 | 0 | 1 | 1 | 1 | 0 | 0 | 0 | 0 | 0 | 0 | 0 |
| **Inocybe grammata** | 0 | 0 | 0 | 0 | 1 | 1 | 0 | 0 | 0 | 0 | 0 | 0 | 0 | 0 | 0 | 0 |
| **Inocybe halophila** R. Heim | 0 | 0 | 0 | 0 | 1 | 0 | 0 | 0 | 0 | 0 | 0 | 0 | 0 | 0 | 0 | 0 |
| **Inocybe langei** R.Heim | 0 | 0 | 0 | 0 | 1 | 0 | 0 | 0 | 0 | 1 | 0 | 0 | 0 | 0 | 0 | 0 |
| **Inocybe maculata** Boud. | 0 | 0 | 0 | 0 | 1 | 1 | 0 | 0 | 0 | 0 | 0 | 0 | 0 | 1 | 1 | 0 |
| **Inocybe mixtilis** Britzelm. | 0 | 0 | 1 | 1 | 1 | 1 | 1 | 1 | 1 | 0 | 0 | 0 | 0 | 1 | 1 | 1 |
| **Inocybe mucronata** R.Heim | 0 | 0 | 0 | 0 | 0 | 0 | 0 | 0 | 0 | 1 | 0 | 0 | 0 | 0 | 0 | 0 |
| **Inocybe napipes** J.E. Lange | 0 | 0 | 0 | 0 | 0 | 0 | 0 | 0 | 1 | 1 | 0 | 0 | 0 | 0 | 0 | 0 |
| **Inocybe patouillardii** Bres. | 0 | 0 | 0 | 0 | 1 | 0 | 0 | 0 | 0 | 0 | 0 | 0 | 0 | 0 | 0 | 0 |
| **Inocybe perbrevis** (Weinm.) Fr. ss. R. Heim | 0 | 0 | 0 | 0 | 1 | 0 | 0 | 0 | 0 | 0 | 0 | 0 | 0 | 0 | 0 | 0 |
| **Inocybe petiginosa** (Fr.: Fr.) Gillet | 0 | 0 | 0 | 1 | 1 | 1 | 0 | 0 | 0 | 0 | 0 | 0 | 0 | 0 | 0 | 0 |
| **Inocybe pisciodora** Donad. & Riousset | 0 | 0 | 0 | 0 | 1 | 0 | 0 | 0 | 0 | 0 | 0 | 0 | 0 | 0 | 0 | 1 |
| **Inocybe pudica** Kühner | 0 | 0 | 0 | 0 | 0 | 0 | 1 | 0 | 1 | 0 | 0 | 0 | 0 | 0 | 0 | 0 |
| **Inocybe tenuicystidiata** var. **meridionalis** Bon | 0 | 0 | 0 | 0 | 1 | 0 | 0 | 0 | 0 | 0 | 0 | 0 | 0 | 0 | 0 | 0 |
| **Inocybe xanthocephala** P.D.Orton | 0 | 0 | 0 | 0 | 0 | 0 | 0 | 0 | 0 | 1 | 0 | 0 | 0 | 0 | 0 | 0 |
| **Laccaria amethystina** Huds. Cooke | 0 | 0 | 1 | 1 | 1 | 1 | 1 | 1 | 1 | 0 | 0 | 0 | 0 | 1 | 0 | 0 |
| **Laccaria bicolor** (Maire) P.D. Orton | 0 | 0 | 0 | 0 | 0 | 0 | 0 | 1 | 1 | 0 | 0 | 0 | 0 | 0 | 0 | 0 |
| **Laccaria proxima** (Boud.) Pat. | 0 | 0 | 0 | 0 | 0 | 0 | 0 | 1 | 1 | 0 | 0 | 0 | 0 | 0 | 0 | 0 |
| **Laccaria pumila** Fayod | 0 | 0 | 0 | 0 | 0 | 0 | 0 | 0 | 0 | 1 | 0 | 0 | 0 | 0 | 0 | 0 |
| **Lactarius acerrimus** J.E. Lange | 0 | 0 | 1 | 1 | 1 | 1 | 0 | 0 | 0 | 0 | 0 | 0 | 0 | 0 | 0 | 0 |
| **Lactarius alpinus** Peck | 0 | 0 | 0 | 0 | 0 | 0 | 0 | 0 | 0 | 0 | 1 | 0 | 0 | 0 | 0 | 0 |
| **Lactarius atlanticus** Bon | 0 | 0 | 0 | 0 | 1 | 0 | 0 | 0 | 0 | 0 | 0 | 0 | 0 | 0 | 0 | 0 |
| **Lactarius aurantiacus** Fr.: Fr. | 0 | 0 | 1 | 1 | 1 | 1 | 1 | 1 | 1 | 0 | 0 | 0 | 0 | 0 | 0 | 0 |
| **Lactarius azonites** (Bull.: Fr.) Fr. | 0 | 0 | 0 | 1 | 1 | 1 | 0 | 0 | 0 | 0 | 0 | 0 | 0 | 0 | 0 | 0 |
| **Lactarius blennius** (Fr.: Fr.) Fr. | 0 | 0 | 0 | 1 | 0 | 0 | 0 | 0 | 0 | 0 | 0 | 0 | 0 | 0 | 0 | 0 |
| **Lactarius bresadolanus** Singer | 0 | 0 | 0 | 0 | 0 | 0 | 1 | 0 | 0 | 0 | 0 | 0 | 0 | 0 | 0 | 0 |
| **Lactarius brunneohepaticus** M.M. Moser | 0 | 0 | 0 | 0 | 0 | 0 | 0 | 0 | 0 | 0 | 1 | 0 | 0 | 0 | 0 | 0 |
| **Lactarius chrysorrheus** Fr. | 0 | 0 | 1 | 0 | 1 | 1 | 0 | 0 | 0 | 0 | 0 | 0 | 0 | 0 | 0 | 0 |
| **Lactarius cistophilus** Bon & trimbach | 1 | 0 | 0 | 0 | 0 | 0 | 0 | 0 | 0 | 0 | 0 | 0 | 0 | 0 | 0 | 0 |
| **Lactarius controversus** (Pers. : Fr.) Pers. | 0 | 0 | 0 | 0 | 0 | 0 | 0 | 0 | 0 | 0 | 0 | 0 | 0 | 0 | 0 | 1 |
| **Lactarius cyathuliformis** Bon | 0 | 0 | 0 | 0 | 0 | 0 | 0 | 0 | 0 | 0 | 0 | 0 | 1 | 0 | 0 | 0 |
| **Lactarius decipiens** Quél. | 0 | 0 | 0 | 0 | 1 | 1 | 0 | 0 | 0 | 0 | 0 | 0 | 0 | 0 | 0 | 0 |
| **Lactarius deliciosus** (L. : Fr.) Gray [ss. lato] | 0 | 0 | 0 | 0 | 0 | 0 | 0 | 1 | 1 | 0 | 0 | 0 | 0 | 0 | 0 | 0 |
| **Lactarius evosmus** Kühner & Romagn. | 0 | 0 | 1 | 1 | 1 | 1 | 0 | 0 | 0 | 0 | 0 | 0 | 0 | 0 | 0 | 1 |
| **Lactarius flavidus** Boud. | 0 | 0 | 0 | 0 | 0 | 0 | 0 | 0 | 0 | 0 | 0 | 0 | 0 | 0 | 1 | 0 |
|  |  |  |  |  |  |  |  |  |  |  |  |  |  |  |  |  |
| **Lactarius fluens** Boud. | 0 | 0 | 0 | 1 | 0 | 0 | 0 | 0 | 0 | 0 | 0 | 0 | 0 | 0 | 0 | 0 |
| **Lactarius fraxineus** Romagn. | 0 | 0 | 0 | 0 | 1 | 1 | 0 | 0 | 0 | 0 | 0 | 0 | 0 | 0 | 0 | 0 |
| **Lactarius fuliginosus** (Fr. : Fr.) Fr. [ss. lat.] | 0 | 0 | 0 | 1 | 0 | 0 | 0 | 0 | 0 | 0 | 0 | 0 | 0 | 0 | 0 | 0 |
| **Lactarius fulvissimus** Romagn. | 0 | 0 | 1 | 1 | 1 | 0 | 0 | 0 | 0 | 0 | 0 | 0 | 0 | 0 | 0 | 0 |
| **Lactarius glyciosmus** (Fr. : Fr.) Fr. | 0 | 0 | 0 | 0 | 0 | 0 | 0 | 0 | 0 | 0 | 0 | 0 | 0 | 1 | 0 | 0 |
| **Lactarius hepaticus** Plowr. | 0 | 0 | 0 | 0 | 0 | 0 | 0 | 1 | 1 | 0 | 0 | 0 | 0 | 0 | 0 | 0 |
| **Lactarius lacunarum** Hora | 0 | 0 | 0 | 0 | 1 | 1 | 0 | 0 | 0 | 1 | 0 | 0 | 0 | 1 | 0 | 1 |
| **Lactarius lepidotus** A.H.Sm. | 0 | 0 | 0 | 0 | 0 | 0 | 0 | 0 | 0 | 0 | 1 | 0 | 0 | 0 | 0 | 0 |
| **Lactarius lilacinus** (Lasch : Fr.) Fr. | 0 | 0 | 0 | 0 | 0 | 0 | 0 | 0 | 0 | 0 | 0 | 0 | 1 | 0 | 0 | 0 |
| **Lactarius luridus** | 0 | 0 | 1 | 1 | 1 | 1 | 0 | 0 | 0 | 0 | 0 | 0 | 0 | 0 | 0 | 0 |
| **Lactarius mairei** Malençon | 0 | 0 | 0 | 0 | 1 | 0 | 0 | 0 | 0 | 0 | 0 | 0 | 0 | 0 | 0 | 0 |
| **Lactarius mediterraneensis** Llistosella & Bellù | 0 | 0 | 0 | 0 | 1 | 1 | 0 | 0 | 0 | 0 | 0 | 0 | 0 | 0 | 0 | 0 |
| **Lactarius mitissimus** (Fr. : Fr.) Fr. | 0 | 0 | 1 | 1 | 1 | 1 | 1 | 1 | 1 | 0 | 0 | 0 | 0 | 0 | 0 | 0 |
| **Lactarius necator** (Bull. : Fr.) Pers. | 0 | 0 | 0 | 0 | 0 | 0 | 0 | 0 | 0 | 0 | 0 | 0 | 0 | 1 | 0 | 0 |
| **Lactarius** **obscuratus** var. **obscuratus** | 0 | 0 | 0 | 0 | 0 | 0 | 0 | 0 | 0 | 0 | 0 | 1 | 1 | 0 | 0 | 0 |
|  |  |  |  |  |  |  |  |  |  |  |  |  |  |  |  |  |
| **Lactarius obscuratus** var. **subalpinus** Basso | 0 | 0 | 0 | 0 | 0 | 0 | 0 | 0 | 0 | 0 | 1 | 0 | 0 | 0 | 0 | 0 |
| **Lactarius omphaliformis** Romagn. | 0 | 0 | 0 | 0 | 0 | 0 | 0 | 0 | 0 | 0 | 0 | 0 | 1 | 0 | 0 | 0 |
| **Lactarius pallidus** (Pers. : Fr.) Pers. | 0 | 0 | 0 | 1 | 0 | 0 | 0 | 0 | 0 | 0 | 0 | 0 | 0 | 0 | 0 | 0 |
| **Lactarius piperatus** (Scop. : Fr.) Pers. | 0 | 0 | 1 | 1 | 0 | 0 | 0 | 0 | 0 | 0 | 0 | 0 | 0 | 0 | 0 | 0 |
| **Lactarius pseudoscrobiculatus** Basso et al. | 1 | 0 | 0 | 0 | 0 | 0 | 1 | 1 | 1 | 0 | 0 | 0 | 0 | 0 | 0 | 0 |
| **Lactarius pyrogalus** (Fr.: Fr.) Fr. | 0 | 0 | 0 | 0 | 0 | 0 | 0 | 0 | 0 | 0 | 0 | 0 | 0 | 0 | 1 | 0 |
| **Lactarius quieticolor** Romagn. | 0 | 0 | 0 | 0 | 0 | 0 | 0 | 0 | 1 | 0 | 0 | 0 | 0 | 0 | 0 | 0 |
| **Lactarius quietus** (Fr.: Fr.) Fr. | 0 | 0 | 1 | 0 | 1 | 1 | 0 | 0 | 0 | 0 | 0 | 0 | 0 | 0 | 0 | 0 |
| **Lactarius resimus** (Fr. : Fr.) Fr. | 0 | 0 | 0 | 0 | 0 | 0 | 0 | 0 | 0 | 0 | 0 | 0 | 0 | 1 | 0 | 0 |
| **Lactarius rufus** (Scop. : Fr.) Fr. | 0 | 0 | 0 | 0 | 0 | 0 | 0 | 1 | 1 | 0 | 0 | 0 | 0 | 1 | 0 | 0 |
| **Lactarius salmonicolor** R. Heim & Leclair | 0 | 0 | 0 | 0 | 0 | 0 | 1 | 0 | 0 | 0 | 0 | 0 | 0 | 0 | 0 | 0 |
| **Lactarius sanguifluus** (Paulet) Fr. | 0 | 0 | 0 | 0 | 0 | 0 | 0 | 1 | 1 | 0 | 0 | 0 | 0 | 0 | 0 | 0 |
| **Lactarius scrobiculatus** (Scop. : Fr.) Fr. | 0 | 0 | 0 | 0 | 0 | 0 | 1 | 0 | 0 | 0 | 0 | 0 | 0 | 0 | 0 | 0 |
| **Lactarius semisanguifluus** R. Heim & Leclair | 0 | 0 | 0 | 0 | 0 | 0 | 0 | 1 | 1 | 0 | 0 | 0 | 0 | 0 | 0 | 0 |
| **Lactarius serifluus** (DC : Fr.) Fr. | 0 | 0 | 0 | 0 | 1 | 1 | 0 | 0 | 0 | 0 | 0 | 0 | 0 | 0 | 0 | 0 |
| **Lactarius subdulcis** (Pers.: Fr.) Gray | 0 | 0 | 0 | 1 | 0 | 0 | 0 | 0 | 0 | 0 | 0 | 0 | 0 | 0 | 0 | 0 |
| **Lactarius subumbonatus** Lindbl. | 0 | 0 | 0 | 1 | 0 | 0 | 0 | 0 | 0 | 0 | 0 | 0 | 0 | 0 | 0 | 0 |
| **Lactarius tesquorum** Malençon | 1 | 0 | 0 | 0 | 0 | 0 | 0 | 0 | 0 | 0 | 0 | 0 | 0 | 0 | 0 | 0 |
| **Lactarius torminosus** (Schaeff. : Fr.) Pers. | 0 | 0 | 0 | 0 | 0 | 0 | 0 | 0 | 0 | 0 | 0 | 0 | 0 | 1 | 0 | 0 |
| **Lactarius trivialis** (Fr.: Fr.) Fr. | 0 | 0 | 0 | 0 | 0 | 0 | 1 | 0 | 0 | 0 | 0 | 0 | 0 | 1 | 0 | 1 |
| **Lactarius uvidus** (Fr.: Fr.) Fr. | 0 | 0 | 1 | 1 | 1 | 1 | 0 | 0 | 0 | 0 | 0 | 0 | 0 | 0 | 0 | 1 |
| **Lactarius vellereus** (Fr. : Fr.) Fr. [ss. lato] | 0 | 0 | 1 | 1 | 1 | 1 | 0 | 0 | 0 | 0 | 0 | 0 | 0 | 0 | 0 | 0 |
| **Lactarius vinosus** Bat. | 0 | 0 | 0 | 0 | 0 | 0 | 0 | 1 | 1 | 0 | 0 | 0 | 0 | 0 | 0 | 0 |
| **Lactarius violascens** (Otto : Fr.) Fr. | 0 | 0 | 1 | 1 | 1 | 1 | 0 | 0 | 0 | 0 | 0 | 0 | 0 | 0 | 0 | 0 |
| **Lactarius volemus** (Fr.: Fr.) Fr. | 0 | 0 | 1 | 1 | 1 | 1 | 0 | 0 | 0 | 0 | 0 | 0 | 0 | 0 | 0 | 0 |
| **Lactarius zonarius** (Bull.) Fr. | 0 | 0 | 0 | 0 | 1 | 1 | 0 | 0 | 0 | 0 | 0 | 0 | 0 | 0 | 0 | 0 |
| **Lactarius zugazae** G. Moreno et al. | 0 | 0 | 0 | 0 | 1 | 0 | 0 | 0 | 0 | 0 | 0 | 0 | 0 | 0 | 0 | 0 |
| **Leccinum aurantiacum** (Bull.: Fr.) S.F. Gray | 0 | 0 | 0 | 0 | 0 | 0 | 0 | 0 | 0 | 0 | 0 | 0 | 0 | 1 | 0 | 0 |
| **Leccinum carpini** | 0 | 0 | 0 | 0 | 0 | 0 | 0 | 0 | 0 | 0 | 0 | 0 | 0 | 0 | 1 | 0 |
| **Leccinum corsicum** (Rolland) Singer | 1 | 0 | 0 | 0 | 0 | 0 | 0 | 0 | 0 | 0 | 0 | 0 | 0 | 0 | 0 | 0 |
|  |  |  |  |  |  |  |  |  |  |  |  |  |  |  |  |  |
| **Leccinum duriusculum** f. *robustum* Lannoy & Estadès | 0 | 0 | 0 | 0 | 0 | 0 | 0 | 0 | 0 | 0 | 0 | 0 | 0 | 0 | 0 | 1 |
| **Leccinum lepidum** (Essette) Bon & Contu | 0 | 0 | 0 | 0 | 1 | 1 | 0 | 0 | 0 | 0 | 0 | 0 | 0 | 0 | 0 | 0 |
| **Leccinum scabrum** (Bull.: Fr.) Gray | 0 | 0 | 0 | 0 | 0 | 0 | 0 | 0 | 0 | 0 | 0 | 0 | 0 | 1 | 0 | 0 |
| **Leccinum versipelle** (Fr.) Snell | 0 | 0 | 0 | 0 | 0 | 0 | 0 | 0 | 0 | 0 | 0 | 0 | 0 | 1 | 0 | 0 |
| **Lindteneria** cf. ***trachyspora*** | 0 | 0 | 0 | 0 | 0 | 0 | 0 | 0 | 0 | 0 | 0 | 1 | 0 | 0 | 0 | 0 |
| **Melanogaster broomeanus** Berk. | 0 | 0 | 0 | 0 | 0 | 0 | 0 | 0 | 0 | 0 | 0 | 0 | 0 | 1 | 0 | 0 |
| **Melanogaster rivularis** P.-A. Moreau & F. Richard | 0 | 0 | 0 | 0 | 0 | 0 | 0 | 0 | 0 | 0 | 0 | 1 | 1 | 0 | 0 | 0 |
| **Melanogaster variegatus** (Vitt.) Tul. & C. Tul. | 0 | 0 | 0 | 0 | 1 | 0 | 0 | 0 | 0 | 0 | 0 | 0 | 0 | 0 | 0 | 0 |
| **Paxillus ammoniavirescens** Contu & Dessi | 0 | 0 | 0 | 0 | 0 | 0 | 0 | 0 | 0 | 0 | 0 | 0 | 0 | 0 | 0 | 1 |
| **Paxillus involutus** (Batsch: Fr.) Fr. | 0 | 0 | 0 | 0 | 0 | 0 | 0 | 1 | 0 | 0 | 0 | 0 | 0 | 0 | 0 | 0 |
| **Paxillus rubicundulus** P.D. Orton | 0 | 0 | 0 | 0 | 0 | 0 | 0 | 0 | 0 | 0 | 0 | 1 | 1 | 0 | 0 | 0 |
| **Peziza badia** Pers. : Fr. | 0 | 0 | 1 | 1 | 0 | 0 | 0 | 1 | 1 | 0 | 0 | 0 | 0 | 0 | 0 | 0 |
| **Peziza succosa** Berk. | 0 | 0 | 1 | 1 | 1 | 1 | 0 | 1 | 1 | 0 | 0 | 0 | 0 | 1 | 1 | 1 |
| **Peziza violacea** Pers. [nomen dubium] | 0 | 0 | 0 | 0 | 0 | 0 | 0 | 1 | 1 | 0 | 0 | 0 | 0 | 0 | 0 | 0 |
|  |  |  |  |  |  |  |  |  |  |  |  |  |  |  |  |  |
| **Phellodon melaleucus** (Fr.. Fr.) P. Karst | 0 | 0 | 0 | 0 | 1 | 0 | 0 | 0 | 0 | 0 | 0 | 0 | 0 | 0 | 0 | 0 |
| **Phellodon niger** (Fr. Fr.) P. Karst. | 0 | 0 | 1 | 1 | 1 | 1 | 1 | 1 | 1 | 0 | 0 | 0 | 0 | 0 | 0 | 0 |
| **Porphyrellus porphyrosporus** (Fr.) Gilbert | 0 | 0 | 0 | 1 | 0 | 0 | 1 | 1 | 0 | 0 | 0 | 0 | 0 | 0 | 0 | 0 |
| **Pyronema omphalodes** | 0 | 0 | 0 | 0 | 0 | 0 | 0 | 1 | 1 | 0 | 0 | 0 | 0 | 0 | 0 | 0 |
| **Ramaria botrytis** | 0 | 0 | 1 | 0 | 1 | 1 | 0 | 0 | 0 | 0 | 0 | 0 | 0 | 0 | 0 | 0 |
| **Ramaria bourdotiana** Maire | 0 | 0 | 0 | 0 | 1 | 0 | 0 | 0 | 0 | 0 | 0 | 0 | 0 | 0 | 0 | 0 |
| **Ramaria quercus-ilicis** Schild | 0 | 0 | 0 | 0 | 1 | 0 | 0 | 0 | 0 | 0 | 0 | 0 | 0 | 0 | 0 | 0 |
| **Rhizopogon corsicus** | 0 | 0 | 0 | 0 | 0 | 0 | 0 | 1 | 0 | 0 | 0 | 0 | 0 | 0 | 0 | 0 |
| **Rhizopogon evadens** A.H. Sm. | 0 | 0 | 0 | 0 | 0 | 0 | 0 | 0 | 1 | 0 | 0 | 0 | 0 | 0 | 0 | 0 |
| **Rhizopogon luteolus** Fr. | 0 | 0 | 0 | 0 | 0 | 0 | 0 | 0 | 1 | 0 | 0 | 0 | 0 | 0 | 0 | 0 |
| **Rhizopogon roseolus** (Corda) Th.M. Fr. | 0 | 0 | 0 | 0 | 0 | 0 | 0 | 1 | 1 | 0 | 0 | 0 | 0 | 0 | 0 | 0 |
| **Rhizopogon roseolus** var. **intermedius** | 0 | 0 | 0 | 0 | 0 | 0 | 0 | 0 | 1 | 0 | 0 | 0 | 0 | 0 | 0 | 0 |
| **Russula acrifolia** Romagn. | 0 | 0 | 0 | 0 | 1 | 1 | 0 | 0 | 0 | 0 | 0 | 0 | 0 | 1 | 0 | 0 |
| **Russula adusta** Fr. | 0 | 0 | 0 | 0 | 0 | 0 | 0 | 1 | 1 | 0 | 0 | 0 | 0 | 0 | 0 | 0 |
| **Russula aeruginea** Lindbl. | 0 | 0 | 0 | 0 | 0 | 0 | 0 | 0 | 0 | 0 | 0 | 0 | 0 | 1 | 0 | 0 |
| **Russula albonigra** Krombh. | 0 | 0 | 0 | 0 | 1 | 0 | 0 | 0 | 1 | 0 | 0 | 0 | 0 | 0 | 0 | 0 |
|  |  |  |  |  |  |  |  |  |  |  |  |  |  |  |  |  |
| **Russula alnetorum** Romagn. | 0 | 0 | 0 | 0 | 0 | 0 | 0 | 0 | 0 | 0 | 1 | 0 | 0 | 0 | 0 | 0 |
| **Russula amara** Kucera | 0 | 0 | 0 | 0 | 0 | 0 | 0 | 1 | 1 | 0 | 0 | 0 | 0 | 0 | 0 | 0 |
| **Russula amoena** Quél. | 0 | 0 | 0 | 0 | 1 | 0 | 0 | 0 | 0 | 0 | 0 | 0 | 0 | 0 | 0 | 0 |
| **Russula amoenicolor** Romagn. | 0 | 0 | 0 | 0 | 1 | 1 | 0 | 0 | 0 | 0 | 0 | 0 | 0 | 0 | 0 | 0 |
| **Russula amoenolens** Romagn. | 0 | 0 | 0 | 0 | 1 | 1 | 0 | 0 | 0 | 0 | 0 | 0 | 0 | 0 | 0 | 0 |
| **Russula anthracina** Romagn. | 0 | 0 | 0 | 0 | 1 | 0 | 0 | 0 | 0 | 0 | 0 | 0 | 0 | 0 | 0 | 0 |
| **Russula anthracina** var. **carneifolia** Romagn. | 0 | 0 | 0 | 0 | 0 | 0 | 1 | 0 | 0 | 0 | 0 | 0 | 0 | 0 | 0 | 0 |
| **Russula aurea** Pers. | 0 | 0 | 0 | 0 | 1 | 0 | 0 | 0 | 0 | 0 | 0 | 0 | 0 | 0 | 0 | 0 |
| **Russula badia** Quél. | 0 | 0 | 0 | 0 | 0 | 0 | 0 | 0 | 1 | 0 | 0 | 0 | 0 | 0 | 0 | 0 |
| **Russula betularum** Hora | 0 | 0 | 0 | 0 | 0 | 0 | 0 | 0 | 0 | 0 | 0 | 0 | 0 | 1 | 0 | 0 |
| **Russula cessans** A. Pearson | 0 | 0 | 0 | 0 | 0 | 0 | 0 | 0 | 1 | 0 | 0 | 0 | 0 | 0 | 0 | 0 |
| **Russula chloroides** Krombh. | 0 | 0 | 0 | 1 | 1 | 1 | 1 | 0 | 1 | 0 | 0 | 0 | 0 | 1 | 0 | 0 |
| **Russula cyanoxantha** (Schaeff.) Fr. | 0 | 0 | 1 | 1 | 1 | 0 | 0 | 0 | 0 | 0 | 0 | 0 | 0 | 0 | 0 | 0 |
| **Russula decipiens** (Singer) Kühner & Romagn. ex Svrček | 0 | 0 | 0 | 0 | 1 | 1 | 0 | 0 | 0 | 0 | 0 | 0 | 0 | 0 | 0 | 0 |
| **Russula delica** Fr. | 0 | 0 | 1 | 0 | 1 | 0 | 0 | 0 | 1 | 0 | 0 | 0 | 0 | 0 | 0 | 0 |
| **Russula delica** var. **trachyspora** | 0 | 0 | 0 | 0 | 1 | 1 | 1 | 1 | 1 | 0 | 0 | 0 | 0 | 0 | 0 | 0 |
|  |  |  |  |  |  |  |  |  |  |  |  |  |  |  |  |  |
| **Russula drimeia** Cooke | 0 | 0 | 0 | 0 | 0 | 0 | 0 | 0 | 1 | 0 | 0 | 0 | 0 | 0 | 0 | 0 |
| **Russula faustiana** Sarnari | 1 | 0 | 0 | 0 | 1 | 0 | 0 | 0 | 0 | 0 | 0 | 0 | 0 | 0 | 0 | 0 |
| **Russula fellea** (Fr.: Fr.) Fr. | 0 | 0 | 0 | 1 | 0 | 0 | 0 | 0 | 0 | 0 | 0 | 0 | 0 | 0 | 0 | 0 |
| **Russula fragilis** (Pers.: Fr.) Fr. | 0 | 0 | 0 | 0 | 1 | 0 | 0 | 0 | 0 | 0 | 0 | 0 | 0 | 0 | 0 | 0 |
| **Russula gigasperma** Romagn. | 0 | 0 | 0 | 0 | 1 | 0 | 0 | 0 | 0 | 0 | 0 | 0 | 0 | 0 | 0 | 0 |
| **Russula graveolens** Romell [ss. lato] | 0 | 0 | 0 | 0 | 1 | 1 | 0 | 0 | 0 | 0 | 0 | 0 | 0 | 0 | 0 | 0 |
| **Russula grisea** Fr. | 0 | 0 | 0 | 1 | 1 | 0 | 0 | 0 | 0 | 0 | 0 | 0 | 0 | 0 | 0 | 0 |
| **Russula heterophylla** (Fr. : Fr.) Fr. | 0 | 0 | 0 | 1 | 0 | 0 | 0 | 0 | 0 | 0 | 0 | 0 | 0 | 0 | 0 | 0 |
| **Russula ilicis** Romagn. et al. | 0 | 0 | 0 | 0 | 1 | 0 | 0 | 0 | 0 | 0 | 0 | 0 | 0 | 0 | 0 | 0 |
| **Russula insignis** Quél. | 0 | 0 | 0 | 0 | 1 | 1 | 0 | 0 | 0 | 0 | 0 | 0 | 0 | 0 | 0 | 0 |
| **Russula integra** (L. : Fr.) Maire | 0 | 0 | 0 | 0 | 0 | 0 | 1 | 0 | 0 | 0 | 0 | 0 | 0 | 0 | 0 | 0 |
| **Russula lepida** Fr. | 0 | 0 | 1 | 1 | 1 | 0 | 0 | 0 | 0 | 0 | 0 | 0 | 0 | 0 | 0 | 0 |
| **Russula littoralis** Romagn. | 0 | 1 | 0 | 0 | 0 | 0 | 0 | 0 | 0 | 0 | 0 | 0 | 0 | 0 | 0 | 0 |
| **Russula luteotacta** Rea | 0 | 0 | 0 | 0 | 1 | 0 | 0 | 0 | 0 | 0 | 0 | 0 | 0 | 0 | 0 | 0 |
| **Russula maculata** Quél. & Roze | 0 | 0 | 0 | 0 | 1 | 0 | 0 | 0 | 0 | 0 | 0 | 0 | 0 | 0 | 0 | 0 |
| **Russula mairei** Singer | 0 | 0 | 0 | 1 | 0 | 0 | 0 | 0 | 0 | 0 | 0 | 0 | 0 | 0 | 0 | 0 |
| **Russula monspeliensis** Sarnari | 1 | 0 | 0 | 0 | 0 | 0 | 0 | 0 | 0 | 0 | 0 | 0 | 0 | 0 | 0 | 0 |
| **Russula mustelina** Fr**.** | 0 | 0 | 0 | 1 | 0 | 0 | 0 | 0 | 0 | 0 | 0 | 0 | 0 | 0 | 0 | 0 |
| **Russula nigricans** (Bull.: Fr.) Fr. | 0 | 0 | 1 | 0 | 0 | 0 | 0 | 0 | 0 | 0 | 0 | 0 | 0 | 0 | 0 | 0 |
| **Russula nitida** Fr. | 0 | 0 | 0 | 0 | 0 | 0 | 0 | 0 | 0 | 0 | 0 | 0 | 0 | 1 | 0 | 0 |
| **Russula ochroleuca** (Pers.) Fr. | 0 | 0 | 1 | 1 | 1 | 1 | 1 | 1 | 1 | 0 | 0 | 0 | 0 | 1 | 0 | 0 |
| **Russula odorata** Romagn. | 0 | 0 | 0 | 0 | 1 | 1 | 0 | 0 | 0 | 0 | 0 | 0 | 0 | 0 | 0 | 0 |
| **Russula pallidospora** Blum ex Romagn. | 0 | 0 | 0 | 1 | 0 | 0 | 0 | 0 | 0 | 0 | 0 | 0 | 0 | 0 | 0 | 0 |
| **Russula pectinatoides** Peck. [ss. Romagn.] | 0 | 0 | 0 | 0 | 1 | 1 | 0 | 0 | 1 | 0 | 0 | 0 | 0 | 0 | 1 | 0 |
| **Russula pelargonia** Niolle | 0 | 0 | 0 | 0 | 1 | 0 | 0 | 0 | 0 | 0 | 0 | 0 | 0 | 0 | 0 | 1 |
| **Russula persicina** Fr. | 0 | 0 | 0 | 0 | 1 | 1 | 0 | 0 | 0 | 0 | 0 | 0 | 0 | 0 | 0 | 1 |
| **Russula poikilochroa** Sarnari | 0 | 0 | 0 | 0 | 1 | 0 | 0 | 0 | 0 | 0 | 0 | 0 | 0 | 0 | 0 | 0 |
| **Russula puellaris** Fr. | 0 | 0 | 0 | 0 | 0 | 0 | 1 | 0 | 0 | 0 | 0 | 0 | 0 | 0 | 0 | 0 |
| **Russula pumila** Rouzeau & Massart | 0 | 0 | 0 | 0 | 0 | 0 | 0 | 0 | 0 | 0 | 0 | 0 | 1 | 0 | 0 | 0 |
| **Russula putida** Sarnari | 0 | 0 | 0 | 0 | 1 | 0 | 0 | 0 | 1 | 0 | 0 | 0 | 0 | 0 | 0 | 0 |
| **Russula risigallina** (Batsch) Sacc. [ss. lato] | 0 | 0 | 0 | 0 | 1 | 0 | 0 | 0 | 0 | 0 | 0 | 0 | 0 | 0 | 1 | 0 |
| **Russula romellii** Maire | 0 | 0 | 0 | 1 | 0 | 0 | 0 | 0 | 0 | 0 | 0 | 0 | 0 | 0 | 0 | 0 |
| **Russula sanguinaria** (Schumach.) Rausch. | 0 | 0 | 0 | 0 | 0 | 0 | 0 | 1 | 1 | 0 | 0 | 0 | 0 | 0 | 0 | 0 |
| **Russula seperina** Dupain | 0 | 0 | 0 | 0 | 1 | 1 | 0 | 0 | 0 | 0 | 0 | 0 | 0 | 0 | 0 | 0 |
| **Russula solaris** Ferdinansen & Winge | 0 | 0 | 0 | 1 | 0 | 0 | 0 | 0 | 0 | 0 | 0 | 0 | 0 | 0 | 0 | 0 |
| **Russula sororia** Fr. | 0 | 0 | 0 | 0 | 1 | 0 | 0 | 0 | 0 | 0 | 0 | 0 | 0 | 0 | 0 | 0 |
| **Russula straminea** Malençon | 0 | 0 | 0 | 0 | 1 | 1 | 0 | 0 | 0 | 0 | 0 | 0 | 0 | 0 | 0 | 0 |
| **Russula subazurea** Bon | 0 | 0 | 0 | 0 | 1 | 1 | 0 | 0 | 0 | 0 | 0 | 0 | 0 | 0 | 0 | 0 |
| **Russula torulosa** Bres. | 0 | 0 | 0 | 0 | 0 | 0 | 0 | 0 | 1 | 0 | 0 | 0 | 0 | 0 | 0 | 0 |
| **Russula turci** Bres. | 0 | 0 | 0 | 0 | 0 | 0 | 0 | 0 | 1 | 0 | 0 | 0 | 0 | 0 | 0 | 0 |
| **Russula tyrrhenica** Sarnari | 1 | 0 | 0 | 0 | 1 | 0 | 0 | 0 | 0 | 0 | 0 | 0 | 0 | 0 | 0 | 0 |
| **Russula velutipes** Velen. | 0 | 0 | 1 | 1 | 1 | 0 | 0 | 0 | 0 | 0 | 0 | 0 | 0 | 0 | 0 | 0 |
| **Russula versicolor** Jul.Schaeff. | 0 | 0 | 0 | 0 | 0 | 0 | 0 | 0 | 0 | 0 | 0 | 0 | 0 | 1 | 0 | 0 |
| **Russula vesca** Fr. | 0 | 0 | 1 | 1 | 1 | 1 | 0 | 1 | 1 | 0 | 0 | 0 | 0 | 0 | 1 | 0 |
| **Russula vinosobrunnea** var. **paraolivacea** M. Bon | 0 | 0 | 0 | 0 | 1 | 0 | 0 | 0 | 0 | 0 | 0 | 0 | 0 | 0 | 0 | 0 |
| **Russula virescens** (Schaeff.) Fr. | 0 | 0 | 1 | 0 | 1 | 1 | 0 | 0 | 0 | 0 | 0 | 0 | 0 | 0 | 0 | 0 |
| **Russula xerampelina** (Schaeff.) Fr. | 0 | 0 | 0 | 0 | 0 | 0 | 0 | 1 | 1 | 0 | 0 | 0 | 0 | 0 | 0 | 0 |
| **Russula zvarae** Velen. | 0 | 0 | 0 | 0 | 1 | 1 | 0 | 0 | 0 | 0 | 0 | 0 | 0 | 0 | 0 | 0 |
| **Sarcodon cyrneus** Maas Geest. | 0 | 0 | 0 | 0 | 1 | 0 | 0 | 0 | 0 | 0 | 0 | 0 | 0 | 0 | 0 | 0 |
| **Sarcodon imbricatus** (L. : Fr.) P. Karst. | 0 | 0 | 0 | 0 | 0 | 0 | 1 | 1 | 0 | 0 | 0 | 0 | 0 | 0 | 0 | 0 |
| **Sarcodon leucopus** (Pers.) Maas Geest. & Nannf. | 0 | 0 | 0 | 0 | 0 | 0 | 0 | 0 | 1 | 0 | 0 | 0 | 0 | 0 | 0 | 0 |
| **Scleroderma areolatum** | 0 | 0 | 1 | 1 | 1 | 1 | 0 | 0 | 0 | 0 | 0 | 0 | 0 | 1 | 0 | 0 |
| **Scleroderma cepa** | 0 | 0 | 0 | 0 | 1 | 0 | 0 | 0 | 0 | 0 | 0 | 0 | 0 | 0 | 0 | 1 |
| **Scleroderma citrinum** Pers.: Pers. | 0 | 0 | 1 | 1 | 0 | 0 | 0 | 0 | 0 | 0 | 0 | 0 | 0 | 0 | 0 | 0 |
| **Scleroderma meridionale** Demoulin & Malençon | 1 | 1 | 0 | 0 | 1 | 1 | 0 | 0 | 1 | 0 | 0 | 0 | 0 | 0 | 0 | 0 |
| **Scleroderma polyrrhizum** Gmel.: Pers. | 0 | 0 | 0 | 0 | 0 | 0 | 0 | 0 | 1 | 0 | 0 | 0 | 0 | 0 | 0 | 0 |
| **Scleroderma verrucosum** (Bull.: Pers.) Pers. | 0 | 0 | 0 | 0 | 1 | 1 | 0 | 0 | 0 | 0 | 0 | 0 | 0 | 0 | 0 | 0 |
| **Scutiger confluens** (Alb. & Schwein. : Fr.) Kotl. & Pouzar | 0 | 0 | 0 | 0 | 0 | 0 | 1 | 1 | 1 | 0 | 0 | 0 | 0 | 0 | 0 | 0 |
| **Scutiger cristatus** (Schaeff.: Fr.) Bondarzew & Singer | 0 | 0 | 0 | 0 | 0 | 0 | 1 | 0 | 0 | 0 | 0 | 0 | 0 | 0 | 0 | 0 |
| **Scutiger ovinus** (Schaeff.: Fr.) Bondarzew & Singer | 0 | 0 | 0 | 0 | 0 | 0 | 1 | 1 | 1 | 0 | 0 | 0 | 0 | 0 | 0 | 0 |
| **Scutiger pescaprae** (Pers.: Fr.) Bondarzew & Singer | 0 | 0 | 0 | 1 | 0 | 0 | 0 | 0 | 0 | 0 | 0 | 0 | 0 | 0 | 0 | 0 |
| **Scutiger subrubescens** Pouzar | 0 | 0 | 0 | 0 | 0 | 0 | 1 | 0 | 0 | 0 | 0 | 0 | 0 | 0 | 0 | 0 |
| **Sphaerosporella brunnea** | 0 | 0 | 0 | 0 | 0 | 0 | 0 | 1 | 1 | 0 | 0 | 0 | 0 | 0 | 0 | 0 |
| **Suillus bellinii** (Inzenga) O. Kuntze | 0 | 0 | 0 | 0 | 0 | 0 | 0 | 0 | 1 | 0 | 0 | 0 | 0 | 0 | 0 | 0 |
| **Suillus bovinus** (L. : Fr.) Roussel | 0 | 0 | 0 | 0 | 0 | 0 | 0 | 1 | 1 | 0 | 0 | 0 | 0 | 0 | 0 | 0 |
| **Suillus collinitus** (Fr.) O. Kuntze | 0 | 0 | 0 | 0 | 0 | 0 | 0 | 1 | 1 | 0 | 0 | 0 | 0 | 0 | 0 | 0 |
| **Suillus granulatus** (L.: Fr.) Roussel | 0 | 0 | 0 | 0 | 0 | 0 | 0 | 1 | 1 | 0 | 0 | 0 | 0 | 0 | 0 | 0 |
| **Suillus luteus** (L.: Fr.) Roussel | 0 | 0 | 0 | 0 | 0 | 0 | 0 | 1 | 1 | 0 | 0 | 0 | 0 | 0 | 0 | 0 |
| **Suillus mediterraneensis** (Jacquet. & Blum) Redeuilh | 0 | 0 | 0 | 0 | 0 | 0 | 0 | 0 | 1 | 0 | 0 | 0 | 0 | 0 | 0 | 0 |
| **Suillus variegatus** (Swartz : Fr.) Richon & Roze | 0 | 0 | 0 | 0 | 0 | 0 | 0 | 1 | 1 | 0 | 0 | 0 | 0 | 0 | 0 | 0 |
| **Thelephora caryophyllea** (Schaeff.: Fr.) Fr. | 1 | 0 | 0 | 0 | 1 | 1 | 0 | 0 | 1 | 0 | 0 | 0 | 0 | 0 | 0 | 0 |
| **Thelephora palmata** Fr.: Fr. | 0 | 0 | 0 | 0 | 0 | 0 | 0 | 0 | 1 | 0 | 0 | 0 | 0 | 0 | 0 | 0 |
| **Thelephora terrestris** Ehrh.: Fr. | 1 | 1 | 1 | 1 | 1 | 1 | 1 | 1 | 1 | 0 | 0 | 0 | 0 | 1 | 1 | 1 |
| **Tomentella chlorina** (Massee) Cunn. | 0 | 0 | 0 | 0 | 1 | 1 | 0 | 0 | 0 | 0 | 0 | 0 | 0 | 0 | 0 | 0 |
| **Torrendia pulchella** Bres. | 0 | 0 | 0 | 0 | 0 | 0 | 0 | 0 | 1 | 0 | 0 | 0 | 0 | 0 | 0 | 0 |
| **Tricharina gilva** | 0 | 0 | 0 | 0 | 0 | 0 | 0 | 1 | 1 | 0 | 0 | 0 | 0 | 0 | 0 | 0 |
| **Tricholoma acerbum** (Bull.: Fr.) Quél. | 0 | 0 | 0 | 0 | 1 | 1 | 0 | 0 | 0 | 0 | 0 | 0 | 0 | 0 | 0 | 0 |
| **Tricholoma albobrunneum** (Pers.: Fr.) Kummer | 0 | 0 | 0 | 0 | 0 | 0 | 0 | 0 | 1 | 0 | 0 | 0 | 0 | 0 | 0 | 0 |
| **Tricholoma album** (Schaeff.: Fr.) Quél. [ss. lato] | 0 | 0 | 0 | 0 | 1 | 0 | 0 | 0 | 0 | 0 | 0 | 0 | 0 | 0 | 0 | 0 |
| **Tricholoma atrosquamosum** (Chev.) Sacc. | 0 | 0 | 0 | 0 | 1 | 1 | 0 | 0 | 0 | 0 | 0 | 0 | 0 | 0 | 0 | 0 |
| **Tricholoma aurantium** (Schaeff.: Fr.) Ricken | 0 | 0 | 0 | 0 | 0 | 0 | 0 | 0 | 1 | 0 | 0 | 0 | 0 | 0 | 0 | 0 |
| **Tricholoma bufonium** (Pers.: Fr.) Gillet | 0 | 0 | 0 | 0 | 1 | 1 | 1 | 0 | 1 | 0 | 0 | 0 | 0 | 0 | 0 | 0 |
| **Tricholoma colossus** (Fr.) Quél. | 0 | 0 | 0 | 0 | 0 | 0 | 0 | 1 | 1 | 0 | 0 | 0 | 0 | 0 | 0 | 0 |
| **Tricholoma columbetta** (Fr.: Fr.) Kummer | 0 | 0 | 1 | 1 | 0 | 0 | 1 | 0 | 0 | 0 | 0 | 0 | 0 | 1 | 0 | 0 |
| **Tricholoma flavovirens** (Pers.: Fr.) Lundell & Nannf. | 0 | 0 | 0 | 0 | 0 | 0 | 0 | 1 | 1 | 0 | 0 | 0 | 0 | 0 | 0 | 0 |
| **Tricholoma focale** (Fr.) Ricken | 0 | 0 | 0 | 0 | 0 | 0 | 0 | 0 | 1 | 0 | 0 | 0 | 0 | 0 | 0 | 0 |
| **Tricholoma fulvum** (Bull.: Fr.) Sacc. | 0 | 0 | 0 | 0 | 0 | 0 | 0 | 0 | 0 | 0 | 0 | 0 | 0 | 1 | 0 | 0 |
| **Tricholoma imbricatum** (Fr.: Fr.) Kummer | 0 | 0 | 0 | 0 | 0 | 0 | 0 | 1 | 1 | 0 | 0 | 0 | 0 | 0 | 0 | 0 |
| **Tricholoma lascivum** (Fr.) Gillet | 0 | 0 | 0 | 1 | 0 | 0 | 0 | 0 | 0 | 0 | 0 | 0 | 0 | 0 | 0 | 0 |
| **Tricholoma matsutake var. nauseosum** | 0 | 0 | 0 | 0 | 1 | 0 | 0 | 0 | 0 | 0 | 0 | 0 | 0 | 0 | 0 | 0 |
| **Tricholoma orirubens** Quél. | 0 | 0 | 0 | 1 | 0 | 0 | 1 | 0 | 0 | 0 | 0 | 0 | 0 | 0 | 0 | 0 |
| **Tricholoma pardinum** (Pers.) Quél. | 0 | 0 | 0 | 0 | 0 | 0 | 1 | 0 | 0 | 0 | 0 | 0 | 0 | 0 | 0 | 0 |
| **Tricholoma pessundatum** (Fr.: Fr.) Quél. | 0 | 0 | 0 | 0 | 0 | 0 | 0 | 0 | 1 | 0 | 0 | 0 | 0 | 0 | 0 | 0 |
| **Tricholoma populinum** J.E. Lange | 0 | 0 | 0 | 0 | 0 | 0 | 0 | 0 | 0 | 0 | 0 | 0 | 0 | 0 | 0 | 1 |
| **Tricholoma portentosum** (Fr.) Quél. | 0 | 0 | 0 | 0 | 1 | 0 | 0 | 1 | 1 | 0 | 0 | 0 | 0 | 0 | 0 | 0 |
| **Tricholoma psammopus** (Kalchbr.) Quél. | 0 | 0 | 0 | 0 | 0 | 0 | 0 | 0 | 1 | 0 | 0 | 0 | 0 | 0 | 0 | 0 |
| **Tricholoma resplendens** (Fr.) Quél. | 0 | 0 | 0 | 0 | 1 | 0 | 0 | 0 | 0 | 0 | 0 | 0 | 0 | 0 | 0 | 0 |
| **Tricholoma saponaceum** (Fr.: Fr.) Kummer | 0 | 0 | 1 | 1 | 1 | 1 | 1 | 1 | 1 | 0 | 0 | 0 | 0 | 0 | 0 | 0 |
| **Tricholoma scalpturatum** (Fr.) Quél. | 0 | 0 | 1 | 1 | 1 | 1 | 1 | 1 | 1 | 1 | 0 | 0 | 0 | 1 | 1 | 1 |
| **Tricholoma sciodes** (Pers.) C.E. Martin | 0 | 0 | 0 | 1 | 0 | 0 | 0 | 0 | 0 | 0 | 0 | 0 | 0 | 0 | 0 | 0 |
| **Tricholoma sejunctum** (Sow.: Fr.) Quél. | 0 | 0 | 0 | 0 | 1 | 1 | 0 | 0 | 0 | 0 | 0 | 0 | 0 | 0 | 0 | 0 |
| **Tricholoma sulphureum** (Bull.: Fr.) Kummer | 0 | 0 | 1 | 1 | 1 | 1 | 0 | 0 | 1 | 0 | 0 | 0 | 0 | 1 | 1 | 0 |
| **Tricholoma terreum** (Schaeff. : Fr.) Kummer [ss. lato] | 0 | 0 | 0 | 0 | 0 | 0 | 0 | 1 | 1 | 0 | 0 | 0 | 0 | 0 | 0 | 0 |
| **Tricholoma ustale** (Fr.: Fr.) Kummer | 0 | 0 | 0 | 1 | 0 | 0 | 0 | 0 | 0 | 0 | 0 | 0 | 0 | 0 | 0 | 0 |
| **Tricholoma ustaloides** Romagn. | 0 | 0 | 0 | 0 | 1 | 0 | 0 | 0 | 0 | 0 | 0 | 0 | 0 | 0 | 0 | 0 |
| **Tubariomyces hygrophoroides** Esteve-Rav., P.A. Moreau & C.E. Hermos. | 1 | 0 | 0 | 0 | 0 | 0 | 0 | 0 | 0 | 0 | 0 | 0 | 0 | 0 | 0 | 0 |
| **Tylopilus felleus** (Bull. : Fr.) P. Karst. | 0 | 0 | 1 | 0 | 1 | 1 | 0 | 1 | 1 | 0 | 0 | 0 | 0 | 0 | 0 | 0 |
| **Xerocomus armeniacus** (Quél.) Quél. | 0 | 0 | 0 | 0 | 1 | 1 | 0 | 0 | 0 | 0 | 0 | 0 | 0 | 0 | 0 | 0 |
| **Xerocomus badius** (Fr.: Fr.) E.J.Gilbert | 0 | 0 | 0 | 0 | 0 | 0 | 1 | 0 | 0 | 0 | 0 | 0 | 0 | 0 | 0 | 0 |
| **Xerocomus chrysenteron** (Bull.) Quél. | 1 | 0 | 1 | 1 | 1 | 1 | 1 | 0 | 0 | 0 | 0 | 0 | 0 | 1 | 0 | 0 |
| **Xerocomus communis** (Bull.) Bon | 0 | 0 | 1 | 1 | 1 | 1 | 1 | 1 | 1 | 0 | 0 | 0 | 0 | 0 | 1 | 1 |
| **Xerocomus ferrugineus** (Schaeff.) Bon | 0 | 0 | 1 | 1 | 1 | 1 | 1 | 1 | 1 | 0 | 0 | 0 | 0 | 1 | 0 | 0 |
| **Xerocomus ichnusanus** Alessio et al. | 0 | 0 | 0 | 0 | 1 | 1 | 0 | 0 | 0 | 0 | 0 | 0 | 0 | 0 | 0 | 0 |
| **Xerocomus pruinatus** (Fr. & Hök) Quél. | 0 | 0 | 0 | 1 | 0 | 0 | 1 | 0 | 0 | 0 | 0 | 0 | 0 | 0 | 0 | 0 |
| **Xerocomus ripariellus** Redeuilh | 0 | 0 | 0 | 0 | 1 | 0 | 0 | 0 | 0 | 1 | 0 | 0 | 1 | 0 | 0 | 1 |
| **Xerocomus roseoalbidus** Alessio & Littini | 0 | 0 | 0 | 0 | 1 | 1 | 0 | 0 | 0 | 0 | 0 | 0 | 0 | 0 | 0 | 0 |
| **Xerocomus rubellus** (Krombh.) Quél. | 0 | 0 | 1 | 0 | 1 | 0 | 0 | 0 | 0 | 0 | 0 | 0 | 0 | 0 | 0 | 0 |
| **Xerocomus subtomentosus** (L. : Fr.) Quél. | 0 | 0 | 0 | 0 | 1 | 1 | 0 | 0 | 0 | 0 | 0 | 0 | 0 | 0 | 0 | 0 |
| **Zelleromyces josserandii** | 0 | 0 | 0 | 0 | 0 | 0 | 0 | 0 | 1 | 0 | 0 | 0 | 0 | 0 | 0 | 0 |

## Supplementary References

## Supplementary References S2: References used to build the dataset.

1. Antonín V, Noordeloos ME. 1997. *A monograph of Marasmius, Collybia and related genera in Europe*. IHW-Verlag, Eching.
2. Bon M, Poumarat S, Fouchier F. 1996. *Marasmiellus trabutii* (Maire) Singer et *M. virgatocutis* Robich, Esteve-Raventós, Moreno. *Bulletin Semestriel de la Fédération des Associations Mycologique Méditerranéennes* 9: 24-33.
3. Borgarino D, Moreau PA. 2007. L’hydne à la joie. Contre-récit d’une découverte mémorable. *Bulletin semestriel de la Fédération des Associations mycologiques méditerranéennes* 31: 85-94.
4. Borgarino D. 2006. Corse île des beautés. *Spécial Champignons Magazine* 52: 40-45.
5. Borgarino D, Moreau PA., Richard F. 2006. Une espèce hydnoïde nouvelle pour la France : *Beenakia mediterranea* comb. nov. *Bulletin de la Société mycologique de France* 121: 187-199.
6. Buyck B, Moreau, PA. 2002. Quelques russules intéressantes récoltées en Corse. *Bulletin Semestriel de la Fédération des Associations Mycologique Méditerranéennes* 21: 5-14.
7. Candusso M. 1996. Una eclatante specie apartenente alla sottosezione Squamulosae (Bat.) Sing., *Hygrocybe corsica* nov. sp. *Rivista Micol.* 39: 11-20.
8. Candusso M. 1997. Tre interesanti Hygrocybe della sottosezione Squamulosae (Bat.) Singer. *Bulletin Semestriel de la Fédération des Associations Mycologique Méditerranéennes* 11: 35-46.
9. Chapon P, Bruzi J, Roux P. 2001. Deux espèces rares récoltées en Corse lors des 14es journées mycologiques de la FAMM. *Bulletin Semestriel de la Fédération des Associations Mycologique Méditerranéennes* 20: 3-9.
10. Chevassut G, Henry R, Tristani A. 1988. Un nouveau cortinaire des chênes verts et arbousiers corses, *Cortinarius* (Phlegm.) *xanthophyllus* (Cke.) Hry, sous-espèce *corsico-amethystinus* ssp. nov. *Bulletin Semestriel de la* *Fédération des Associations Mycologique Méditerranéennes* 19: 43-44.
11. Corriol G. 2005. Contribution à la connaissance des champignons de l’étage thermoméditerranéen corse. *Bulletin Semestriel de la* *Fédération des Associations Mycologique Méditerranéennes* 27: 3-20.
12. Corriol G. 2006. *Marasmius mediterraneus* sp. nov. Une espèce nouvelle de la section Sicci. *Bulletin de la Société mycologique de France* 122: 97-106
13. Eyssartier G. 2002. *Entoloma ursulae* Noordel. en Corse. *Bulletin Semestriel de la* *Fédération des Associations Mycologique Méditerranéennes* 22: 39-45.
14. Eyssartier G. 2003. Révision de la clé des *Entoloma* « bleus » publiée dans le précédent numéro et compléments. *Bulletin Semestriel de la Fédération des Associations Mycologique Méditerranéennes* 23: 43-46, 48.
15. Eyssartier G, Moreau PA. 2001. Notes sur quelques espèces intéressantes de basidiomycètes récoltées en Corse. *Bulletin Semestriel de la Fédération des Associations Mycologique Méditerranéennes* 20: 11-22.
16. Fidalgo O. 1974. *Osmoporus odoratus* and its European varieties. *Rickia* 6: 27-61.
17. Jülich W. 1984. Die Nichtblätterpilze, Gallertpilze und Bauchpilze. Stuttgart New York, Germany: G. Fischer
18. Lutz L. 1901. Champignons récoltés en Corse pendant les mois de juin et de juillet 1900. *Bulletin de la Société mycologique de France* 17: 121-122.
19. Maire R, Dumée P, Lutz L. 1903. Prodrome d’une flore mycologique de la Corse. In : collectif – Session extraordinaire en Corse, mai-juin 1901. *Bulletin de la Société botanique de France* 48: 179-247.
20. Malaval JC. 2005. *Torrendiella ciliata*, Ascomycetes trouvé en France (Corse et Provence) lors des Journées mycologiques en 2004. *Bulletin Semestriel de la Fédération des Associations Mycologique Méditerranéennes* 28: 41-46.
21. Malençon G, Bertault R. 1975. *Flore des champignons supérieurs du Maroc. Tome 1.* Rabat, Maroc: Faculté des sciences de Rabat.
22. Maurice JP. 2001. *Gymnopilus corsicus* Romagnesi. *Bulletin Semestriel de la Fédération des Associations Mycologique Méditerranéennes* 19: 27-32.
23. Mayor E, Terrier C. 1957. Excursions mycologiques en Corse. *Revue de Mycologie (Paris)* 22: 113-129.
24. Mayor E, Terrier C. 1959. Nouvelles excursions mycologiques en Corse. *Revue de Mycologie (Paris)* 24: 386-418.
25. Mayor E, Viennot-Bourgin G. 1950. Contribution à l’étude des micromycètes de Corse. *Revue de Mycologie (Paris)* 15: 80-118.
26. Meyer M, Poulain M. 2001. Les myxomycètes de Corse. *Bulletin Semestriel de la Fédération des Associations Mycologique Méditerranéennes* 19: 5-12.
27. Michau G. 1988. *Contribution à l’inventaire des champignons supérieurs de la Corse*. Ajaccio, France: Association des Amis du Parc Naturel Régional de la Corse.
28. Michel H, Duhem B, Trichies G. 2006. Nouveau regard sur Poria millavensis. *Bulletin de la Société mycologique de France* 121: 29-46.
29. Milleliri AM. 1985. *Les champignons supérieurs de Corse (inventaire – comestibilité – toxicité – écologie).* Phd thesis, Université Marseille I, France.
30. Moënne-Loccoz P, Reumaux P. 1990. *Atlas des Cortinaires*, pars II. Vézeronce-Curtin, France: Fédération Mycologique Dauphiné-Savoie.
31. Moreau PA, Contu M. 2007. Une espèce remarquable de l’étage thermoméditerranéen de Corse et de Sardaigne : *Tricholosporum cossonianum* (Maire) comb. nov. *Bulletin Semestriel de la Fédération des Associations Mycologique Méditerranéennes* 32: 41-52.
32. Moreau PA, Garcia G. 2005. *Alnicola salabertii*, espèce nouvelle mycorhizique d'*Alnus cordata*, et deux autres *Alnicola* microsporés. *Bulletin de la Société mycologique de France* 120: 273-292.
33. Moreau PA, Corriol G, Borgarino D, Lavoise C. 2007. Contribution à la connaissance des champignons de l’étage thermoméditerranéen corse III. Description de quelques espèces rares ou nouvelles. *Bulletin Semestriel de la* *Fédération des Associations Mycologique Méditerranéennes* 31: 33-84.
34. Moreau PA, Corriol G, Borgarino D, Aubel P, Lavoise C, Richard F, Selosse MA. 2007. Contribution à la connaissance des champignons de l’étage thermoméditerranéen corse II. *Bulletin Semestriel de la Fédération des Associations Mycologique Méditerranéennes* 31: 9-31.
35. Moreau PA, Roux P, Mascarell G. 1999. Une monographie du genre *Lentinellus* en Europe. *Bulletin de la Société mycologique de France* 115: 5-117.
36. Moyersoen B, Demoulin V. 1996. Les Gastéromycètes de Corse : taxinomie, écologie, chorologie. *Lejeunia nouvelle série* 152: 1-128.
37. Neville P. 1988. Contribution à l’inventaire des Macromycètes de la Corse I. *Bulletin Semestriel de la* *Fédération des Associations Mycologique Méditerranéennes* 3 : 26-51.
38. Neville P. 1992. Quelques Basidiomycotina méditerranéens rares ou intéressants I. *Bulletin Semestriel de la* *Fédération des Associations Mycologique Méditerranéennes* 1 : 27-31.
39. Neville P, Bertea P, Alesandri J, Chabrol J, Lafuente G, Tristani A. 1987. Contribution à l’inventaire des Macromycètes de la Corse. *Bulletin Semestriel de la Fédération des Associations Mycologique Méditerranéennes* 2: 21-39.
40. Norstedt G. 1999. Contribution à la connaissance des polypores des forêts de pin laricio (*Pinus nigra subsp. laricio* Poiret). *Bulletin Semestriel de la Fédération des Associations Mycologique Méditerranéennes* 16: 13-21.
41. Norstedt G, Bader P, Ericson L. 2001. Polypores as indicators of conservation value in Corsican pine forests. *Biological Conservation* 99: 347-354.
42. Pieri M, Rivoire B. 1992. Contribution à l’inventaire des Aphyllophoromycetideae et Heterobasidiomycetes de la Corse I. Liste des espèces récoltées lors des IVe rencontres. *Bulletin Semestriel de la* *Fédération des Associations Mycologique Méditerranéennes* 2: 5-10.
43. Pieri M, Rivoire B. 1994. Contribution à l’inventaire des Aphyllophoromycetideae et Heterobasidiomycetes de la Corse II. Hydnées et polypore signalés en Corse par divers auteurs. *Bulletin Semestriel de la* *Fédération des Associations Mycologique Méditerranéennes* 5: 15-19.
44. Pieri M, Rivoire B. 2003. Les premières mentions de *Perenniporia ochroleuca* en Europe et sur le pourtout méditerranéen. Inventaire des supports ligneux colonisés par cette espèce en Europe. *Bulletin Semestriel de la Fédération des Associations Mycologique Méditerranéennes* 24: 29-36.
45. Pieri M. 1993. Un polypore rare : *Spongipellis delectans* (Peck) Murr. *Bulletin semestriel de la Fédération des Associations mycologiques méditerranéennes* 4: 40-45.
46. Reid DA. 1968. Spring fungi in Corsica. *Revue de Mycologie (Paris)* 33: 3-27.
47. Reid DA. 1969. Spring fungi in Corsica (suite). *Revue de Mycologie (Paris)* 33: 341-368.
48. Reid DA. 1969. Spring fungi in Corsica (suite). *Revue de Mycologie (Paris)* 33: 232-267.
49. Richard F, Alesandri J. 2005. Les champignons de Corse. In : Sabiani A. éds. *Encyclopedia Corsicae Volume I: Géologie, la mer, la terre*. Bastia, France: Dumane, p572-599.
50. Richard F, Selosse MA. 2006. Phénologie des fructifications fongiques dans une forêt corse : le cas de la forêt de chêne vert (*Quercus ilex* L.) du Fango. *Bulletin Semestriel de la Fédération des Associations Mycologique Méditerranéennes* 29: 5-13.
51. Richard F, Hugot L, Moreau PA. 2006. Des mycologues tout-terrain. *Spécial Champignons Magazine* 52: 46-49.
52. Richard F, Moreau PA, Selosse MA, Gardes M. 2004. Diversity and fruiting patterns of ectomycorrhizal and saprobic fungi in an old-growth Mediterranean forest dominated by *Quercus ilex* L. *Canadian Journal of Botany* 82: 1711-1729.
53. Rolland L. 1898. Excursions mycologiques dans le midi de la France et notamment en Corse, en octobre 1897. *Bulletin trimestriel de la Société mycologique de France* 14: 85.
54. Romagnesi H. 1973. Le congrès de la Société mycologique de France à Ajaccio (7-15 octobre 1972). *Bulletin de la Société mycologique de France* 89: 95-100.
55. Romagnoli M. 1863. *Collection des champignons de la Corse, récoltés, dessinés et coloriés par M. Romagnoli, ancien pharmacien et naturaliste*. Unpublished manuscript, Museum of Ajaccio.
56. Roux P, Fouchier F. 1996. Contribution à l’étude des Coprinaceae de la zone méditerranéenne (1re note). *Coprinus trisporus* Kemp, Watling. *Bulletin Semestriel de la Fédération des Associations Mycologique Méditerranéennes* 10: 3-9.
57. Roux P. 2006. *Mille et un champignons.* P. Roux ed., Sainte-Sigolène, France.
58. Rouxel J, Coulom M, Chapon P, Roux P, Fouchier F. 2001. *Marasmius hellebori-corsici* Romagnesi, Un taxon endémique corse. *Bulletin Semestriel de la* *Fédération des Associations Mycologique Méditerranéennes* 20: 39-44.
59. Roy, M., Rochet, J., Manzi, S., Jargeat, P., Gryta, H., Moreau, P.-A., and Gardes, M. (2013). What determines Alnus-associated ectomycorrhizal community diversity and specificity? A comparison of host and habitat effects at a regional scale. *New Phytol* 198, 1228–1238. doi:10.1111/nph.12212.
60. Schopfer J. 2006. Récolte corse d’un taxon très rare : *Conocybe fiorii* (Sacc.) Watling. *Bulletin Semestriel de la Fédération des Associations Mycologique Méditerranéennes* 29: 25-30.
61. Tristani A. 1994. Un cortinaire spectaculaire : *Cortinarius xanthophyllus ssp. corsico-amethystinus*. *Bulletin Semestriel de la Fédération des Associations Mycologique Méditerranéennes* 6: 28-29.

## Supplementary Methods

## Supplementary Methods S3: Species included in the analysis (see also Fig. S4)

We constructed a network based on 23 known ectomycorrhizal plant species present in Corsica (Gamisans 1991; Jeanmonod and Gamisans 2007). We excluded mycoheterotrophic and mixotrophic plants (orchids and Pyroleae; Selosse and Roy 2009) from the analysis because of the atypical physiology of their relationship with ECM fungi. Four additional plant species were excluded from the analysis because of their very low frequency (*Pinus pinea,* *Populus canescens*, *Pinus halepensis* and *Quercus robur* subsp. *robur*) or because their ECM fungal communities are insufficiently documented (*Arbutus unedo*, *Fumana laevipes*, *Fumana thymifolia*, *Ostrya carpinifolia*, *Populus tremula*, *Quercus humilis* subsp. *humilis* and *Quercus petraea* subsp. *petraea*).

*Salix* species (3 species) co-occur in Corsica and their local ECM communities have not been critically analyzed using molecular tools, and the same for species of the genus *Cistus* (4 species). Therefore we conservatively considered them as a single taxon in the analysis (*Salix ssp* and *Cistus ssp*). Finally, four recently introduced species (*Eucalyptus globulus*, *Larix decidua*, *Pseudotsuga menziesii* and *Populus nigra*) were excluded from the analysis.

Hence the 16 resulting synthetic plant species (hereafter called plant species) included all the tree species dominating the forest stages of plant ecological successions in Corsica (Gamisans 1991). They represented all forest ecosystems from sea level (sclerophyllous oak forests) to the upper altitudinal tree limit. These 16 species constitute the plant nodes of our plant-fungi bipartite network.

For macromycetes, four ECM fungal groups, representing 411 species out of 610 ECM fungal species recorded in Corsica (Table S1), were included in the network analysis. We retained all groups for which were available at the species level (i) a well-resolved taxonomic treatment, and (ii) reliable data on host specificity within Corsica based on published records (Supplementary references S2). Hence, the two major Sebacinaceae and Thelephoraceae ECM groups were excluded from the analyses (Horton and Bruns 2001) because their inconspicuous spore-bearing structures are only sporadically recorded in field surveys. Ascomycetes were also excluded for lack of information concerning their associated hosts but also because of new data about the biotrophic status of various Pezizomycetes (Tedersoo et al. 2013), and because the main ECM groups are inconspicuous (Pyronemataceae, hypogeous Eurotiales and Pezizales) or unreliably identified due to taxonomic difficulties (*Helvella*, *Peziza*, etc.). Anyhow, the plant-plant projected network would only get denser (more links or links with a stronger weight) by taking into account more fungal species, hence our results are more significant with the stringent selection of fungal species described above.

For the 411 selected fungal species, association with the 16 synthetic plant species was compiled as follows. Validated associations between plants and fungi were extracted from reports published in mycological journals by expert mycologists (deduced from unambiguous field observations associated with herbaria specimens and georeferenced sampling locations; see supplementary references) and molecular studies (e.g. Richard et al*.* 2004, 2005; Moreau et al*.* 2011; Rochet et al*.* 2011), completed by general ecological information on species given in Courtecuisse and Duhem (2000). Herbarium collections (published or not, especially from the Lille University herbarium LIP, France) related to field observations were systematically used to validate the taxonomic identity of fungi.

## Supplementary Figures

## Figure S4: Workflow of data filtering to build the dataset

## Figure S5: Equivalent of figure 3 in a fungal perspective

## Figure S6: Distributions of plants’ networks metrics

## Figure S7: Matrix representation of the plant projected network. The dotted lines separate early-stage plants (e.g. *Salix spp*) and late-stage plants (e.g. *Quercus suber*).

## Figure S8: Enhanced plants’ networks metrics comparisons between early stage and late stage plants species

## Figure S9: Average distance (Jaccard) between fungal communities regardless their host ecology.


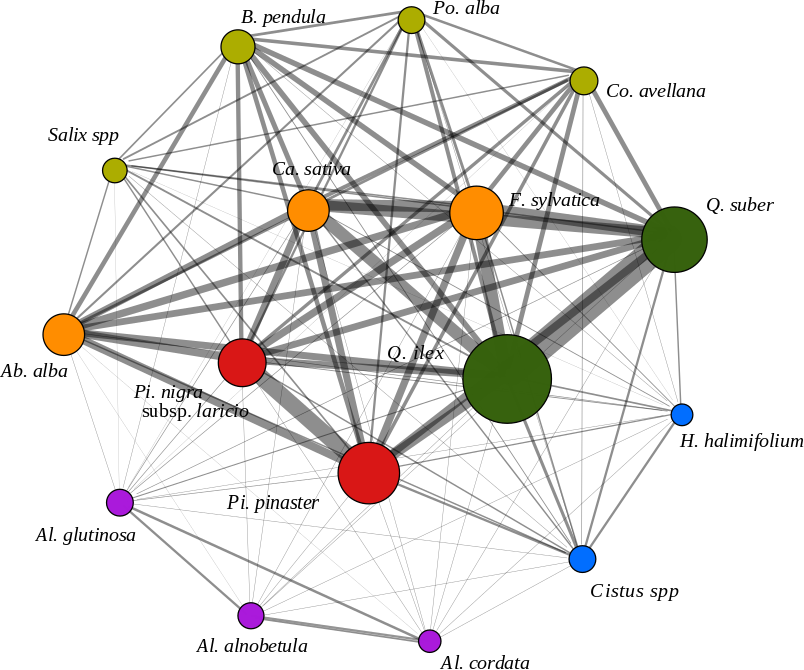


Figure S10: Plant projected weighted network. Each circle is a plant. The line width of each link is proportional to the number of shared ECM fungal partners between plants. Colors indicate modules also shown in figure 1. This figure was obtained using the software GEPHI and The Fruchterman Reinold algorithm.

## Supplementary Script

## Supplementary Material S11: R function to calculate networks metrics used in this paper

A is a bipartite interaction matrix.

net.bip <- function (A = NULL) {

#---------------

# Calculation of the projected network matrix

B <- A%*%t(A)

C <- t(A)%*%A

#---------------

# I is the identity matrix

I.B <- diag(1, dim(B)[1])

I.C <- diag(1, dim(C)[1])

B <- B-diag(B)*I.B

C <- C-diag(C)*I.C

#---------------

#k: number of partners (r for row and c for column)

k_r <- rowSums(A)

k_c <- colSums(A)

#---------------

#s: strength of nodes in the projected network (weighted links)

s_r <- colSums(B)

s_c <- colSums(C)

#---------------

#l: number of partners in the projected network (unweighted links)

l_r <- colSums(B>=1)

l_c <- colSums(C>=1)

#__________________________

# c: Average number of partners of the partners of a given species

c_r <- 1+s_r/k_r

c_c <- 1+s_c/k_c

#__________________________

# Create result list

res <- list()

res$A <- A

res$B <- B

res$C <- C

res$k_r <- k_r

res$k_c <- k_c

res$l_r <- l_r

res$l_c <- l_c

res$s_r <- s_r

res$s_c <- s_c

res$c_r <- c_r

res$c_c <- c_c

return(res)

}

**Results:**

$A is the initial bipartite interaction matrix

$B and $C are the two projected matrices (square symmetric matrices)

$k is the number of partners ($k_r for row and $k_c for column)

$l is the number of partners in the projected network (unweighted links, $l_r for row (*i.e.* based on projected network $B) and $l_c for column (*i.e.* based on projected network $C))

$s is the nodes strength in the projected network (weighted links; $s_r for row and $s_c for column)

$c is the average number of partners of the partners of a given species ($c_r for row and $c_c for column)

For example, if the initial bipartite matrix A is a matrix with plants in columns and fungi in rows; B gives the fungal projected network and C the plant projected network, k_r is the number of plant partners of fungal taxa and k_c the number of fungal partners of plants taxa.
